# Ultraflexible and transparent electroluminescent skin for real-time and super-resolution imaging of pressure distribution

**DOI:** 10.1038/s41467-020-14485-9

**Published:** 2020-01-31

**Authors:** Byeongmoon Lee, Ji-Young Oh, Hyeon Cho, Chul Woong Joo, Hyungsoo Yoon, Sujin Jeong, Eunho Oh, Junghwan Byun, Hanul Kim, Seunghwan Lee, Jiseok Seo, Chan Woo Park, Sukyung Choi, Nae-Man Park, Seung-Youl Kang, Chi-Sun Hwang, Seong-Deok Ahn, Jeong-Ik Lee, Yongtaek Hong

**Affiliations:** 1 Department of Electrical and Computer Engineering, Inter-University Semiconductor Research Center (ISRC), Seoul National University, 1 Gwanak-ro, Gwanak-gu, Seoul 08826 Republic of Korea; 2 Reality Device Research Division, ICT Materials & Components & Research Laboratory, Electronics & Telecommunications Research Institute (ETRI), Daejeon, 34129 Republic of Korea; 3 Department of Mechanical and Aerospace Engineering, Institute of Advanced Machines and Design, Seoul National University, 1 Gwanak-ro, Gwanak-gu, Seoul 08826 Republic of Korea; 4 Soft Robotics Research Center (SRRC), Seoul National University, 1 Gwanak-ro, Gwanak-gu, Seoul 08826 Republic of Korea

**Keywords:** Electrical and electronic engineering, Sensors and biosensors, Nanowires

## Abstract

The ability to image pressure distribution over complex three-dimensional surfaces would significantly augment the potential applications of electronic skin. However, existing methods show poor spatial and temporal fidelity due to their limited pixel density, low sensitivity, or low conformability. Here, we report an ultraflexible and transparent electroluminescent skin that autonomously displays super-resolution images of pressure distribution in real time. The device comprises a transparent pressure-sensing film with a solution-processable cellulose/nanowire nanohybrid network featuring ultrahigh sensor sensitivity (>5000 kPa^−1^) and a fast response time (<1 ms), and a quantum dot-based electroluminescent film. The two ultrathin films conform to each contact object and transduce spatial pressure into conductivity distribution in a continuous domain, resulting in super-resolution (>1000 dpi) pressure imaging without the need for pixel structures. Our approach provides a new framework for visualizing accurate stimulus distribution with potential applications in skin prosthesis, robotics, and advanced human-machine interfaces.

## Abstract (web summary)

Electronic skin that spatially maps pressure distribution through imaging shows limited performance despite improvements to data acquisition. Here, the authors report ultraflexible, transparent electroluminescent skin capable of high-resolution imaging of pressure distribution over 3D surfaces.

## Introduction

Electronic skin (e-skin) seamlessly interfaces living organisms with computers, realizing novel applications such as health monitoring^1,2^, medical implants^3,4^, and user interfaces for augmented reality (AR)^5,6^. Intimate and conformable access to the sources of physical and biological signals has been achieved by adopting soft^7,8^ or ultrathin^9,10^ forms of electronic sensors that can detect pressure^11–14^, strain^15,16^, temperature^17^, and chemical substances^18^. Beyond detection at a specific point, spatial mapping of a mechanical stimulus, such as pressure, over complex three-dimensional (3D) surfaces would greatly augment the potential applications of e-skin such as skin prosthesis^19,20^ and artificial sensory systems for soft robotics^21,22^, and offer potential opportunities to verify numerical analysis for mathematical physics^23^. In this regard, previous works have tried to map spatial pressure by employing a deformable sensor array with a matrix design^24–27^. However, they cannot fully comprehend this analogue signal because of low pixel density or crosstalk among pixels. In particular, unlike other stimuli, spatial pressure could be severely distorted by the device structure itself, such as thick or uneven sensing layers and structural inhomogeneity between the pixel and non-pixel areas. Moreover, bulky electrical wires for data acquisition cause unstable operation of the devices on complex surfaces, thereby impeding high-resolution pressure mapping.

Recently, methods for the direct imaging of spatial pressure with “human-/machine-readable visual output” have been proposed to avoid this complicated data acquisition process^28–32^. When incorporated with pre-established digital imaging technology, such devices can wirelessly transmit the measured information to the machine. Enabling technologies include an integrated active matrix of pressure sensors and organic light-emitting devices^28^, the mechanoluminescent effect of inorganic phosphors^32^, and a piezoelectric nanowire light-emitting diode (LED) array^29–31^. Although they showed promising concepts and applications, these existing methods suffer from limited spatial resolution, respond to only dynamic pressure change, or show extremely low sensitivity. In particular, piezoelectric fluorescent materials have great potential for pressure sensing and imaging without an external power source^32^; however, they respond to only dynamic pressure change. Although pressure-imaging devices using piezoelectric nanowire LEDs have achieved high pixel resolution^29–31^, their extremely low sensitivity and low fill factor degrade the image quality and hinder their practical applications. Furthermore, most of the previous works cannot offer pressure imaging over arbitrary surfaces due to their limited conformability. In other words, there have not yet been fully conformable electronics capable of imaging real-time pressure distribution in a practical range (<1 MPa) with high spatial and temporal fidelity at the same time. Realization of such devices demands comprehensive development of new materials and manufacturing methods for high-sensitivity pressure sensors and appropriate device design for capturing undistorted spatial pressure with high-information density.

Here, we report an ultraflexible and transparent electroluminescent skin that autonomously displays an unprecedented high-fidelity “continuous image” of pressure distribution in real-time. The device consists of two ultrathin films in contact: a transparent pressure-sensing film and quantum-dot-based electroluminescent film. Especially, for the ultrathin and transparent pressure-sensing film, we develop a solution-processable cellulose/nanowire nanohybrid network (CNN), whose unique nanostructured surface morphology enables high transmittance (~80%), ultrahigh sensitivity (>5000 kPa^−1^), great linearity over a wide working range, and a fast response time (<1 ms). Because the two ultrathin films perfectly conform to each contact object, the CNN can transduce high-fidelity spatial pressure into conductivity distribution in a continuous domain, resulting in super-resolution (>1000 dpi) analogue imaging of pressure distribution without the need for pixel structures. Due to the high sensitivity and superior spatial resolution, our device even clearly visualizes pressure distribution arising from micro-textures of soft bodies such as a fingerprint. We further construct real-time smart touch interfaces that can identify the user as well as touch force and location by integrating our electroluminescent skin with a digital imaging system. Our approach provides a new framework for visualizing accurate stimulus distribution with potential applications in skin prosthesis, robotics and advanced human-machine interfaces.

## Results

### Ultraflexible and pressure-sensitive electroluminescent skin

**Fig. 1:**
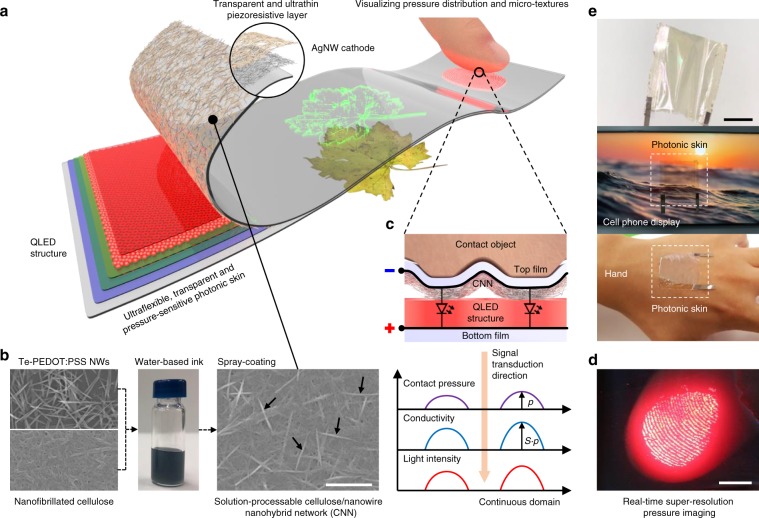
Ultraflexible, transparent and pressure-sensitive electroluminescent skin. **a** Conceptual illustration of an ultraflexible, transparent and pressure-sensitive photonic skin and its device structure. **b** Fabrication scheme and morphology of the cellulose/nanowire nanohybrid network (CNN). The left scanning electron microscopy (SEM) images show tellurium-poly(3,4-ethylenedioxythiophene):poly(styrenesulfonate) (Te-PEDOT:PSS) nanowires and nanofibrillated cellulose, respectively. The middle photograph shows the water-based nanocellulose/Te-PEDOT:PSS nanowire hybrid ink. The right SEM image shows the spray-coated CNN. Dense cellulose nanofibres encircling each Te-PEDOT:PSS nanowire in the CNN, forming a nanostructured surface morphology. Some Te-PEDOT:PSS nanowires are indicated by arrows. Scale bar, 1 μm. **c** Schematic illustration of super-resolution and high-contrast pressure imaging where a rugged soft contact object touches the pressure-sensitive photonic skin on a flat surface. As the ultrathin top film conforms to the contact object, the CNN efficiently transduces the contact pressure into the conductivity distribution between a common cathode and an electron transport layer (ETL) with a high linear conversion factor of *S* (sensitivity of the CNN) in a continuous spatial domain, resulting in electroluminescent analogue imaging of the contact pressure. **d** Optical photograph of the real-time and super-resolution pressure imaging resulting when a finger touched the photonic skin. Scale bar, 5 mm. **e** Optical photographs of the fabricated photonic skin. Because the total thickness of the device is ~3 µm, it can be laminated on various three-dimensional (3D) surfaces, such as a curved display and human skin, conformably and imperceptibly in terms of the senses of both touch and vision. Scale bars, 1 cm.

Fig. 1a illustrates the concept and structure of the pressure-sensitive electroluminescent skin. The device has a notably straightforward and pixel-less structure where a top film coated with a cathode and CNN as a transparent piezoresistive layer is in contact with an electron transport layer (ETL) of a quantum-dot light-emitting diode (QLED) on a bottom film. Unique morphology and superior piezoresistive performance of the CNN play a critical role in the design and performance of the electroluminescent skin. Unlike previous cellulose-based pressure sensors, where microfibres are coated with conductive nanowires^33–36^, our CNN features dense cellulose nanofibres encircling each tellurium-poly(3,4-ethylenedioxythiophene):poly(styrenesulfonate) (Te-PEDOT:PSS) nanowire, forming a nanostructured surface morphology (Fig. 1b), which enables ultrathin design (~1-µm-thick) with ultrahigh sensor sensitivity (>5000 kPa^−1^) and a wide working range. Superfine pressure imaging is enabled by incorporation of the high sensitivity of the CNN and the high conformability of the two ultrathin films (~1-µm-thick). When two 3D objects make contact with both sides of the electroluminescent skin, the top and bottom films conform to each contact object. The CNN on the top film then touches the top surface of the ETL, forming conductivity distribution between the cathode and the ETL linearly proportional to the pressure distribution with a high conversion factor of *S* (sensitivity of the CNN) in a continuous domain (Fig. 1c). Because the two films are sufficiently thin so they can perfectly conform to each surface of the contact objects, minimal distortion occurs in the pressure distribution between the two objects, and therefore the conductivity distribution as well. This spatially patterned conductivity results in fast (<1 ms) and super-resolution (>1000 dpi) electroluminescent analogue imaging under a bias voltage where the local light intensity quantifies the local pressure with high sensitivity and linearity (Fig. 1d). Because all spatial signals from pressure to light intensity are transduced in an analogue manner, our device visualizes pressure distribution without the need for pixel structures.

The fabrication procedure comprises facile and low-cost solution processes, showing high potential for practical applications. A QLED structure excluding a top cathode was formed on a bottom colourless polyimide (PI) film (~1-µm-thick) by a well-controlled solution process. A silver nanowire (AgNW) transparent electrode as a common cathode and a CNN were sequentially deposited on a top colourless PI film (~1-µm-thick) by spin-coating and spray-coating, respectively. The top film was in turn laminated on the bottom film. The detailed fabrication process and the device structure are depicted in the Methods and Supplementary Fig. 1. After delamination from the supporting glass substrate, an ultraflexible and transparent two-terminal device was obtained, which is referred to as a pressure-sensitive photonic skin or photonic skin for short (Fig. 1e). The total thickness of our device was ~3 µm, and the transmittance in the visible spectral region was ~60% (Supplementary Fig. 2). The device can be laminated on any type of surface, such as a curved display and human skin, conformably and imperceptibly in terms of the senses of both touch and vision.

### Piezoresistive characterization of the CNN

Conventional piezoresistive pressure sensors employing composites of conductive fillers and insulating rubbers usually suffer from low sensitivity, slow response time, and significant hysteresis due to the mechanical instability of the rubber matrices^5,37^. Alternatively, conductive nanomaterials impregnated in porous structures in contact with electrodes show high sensitivity and fast response^33–36,38–44^ but cannot offer viability due to impractical methods requiring micro-fabricated^42–44^ or pre-established structures such as tissue paper^33–35^ and a sponge^39^. In this work, we develop a water-based nanocellulose/Te-PEDOT:PSS nanowire hybrid ink for facile and reliable large-area fabrication of ultrathin and transparent piezoresistive layers that can be readily integrated into thin film devices such as our photonic skin. Aqueous dispersions of Te-PEDOT:PSS nanowires and nanofibrillated cellulose were mixed into the solution-processable ink (details in Methods). Uniform and ultrathin random networks of the nanowires and the nanofibres were deposited by spray-coating this aqueous mixture (Fig. 1b and Supplementary Fig. 3). The main advantage of our method is that the thickness, transparency and initial conductivity of the CNN can be easily controlled by the volume ratio of the mixture and spraying conditions. The spray-coated CNN shows variable transmittance as a function of the initial sheet resistance from 60% at 50 kΩ sq^−1^ to 82% at 270 kΩ sq^−1^ (Supplementary Fig. 4).

**Fig. 2:**
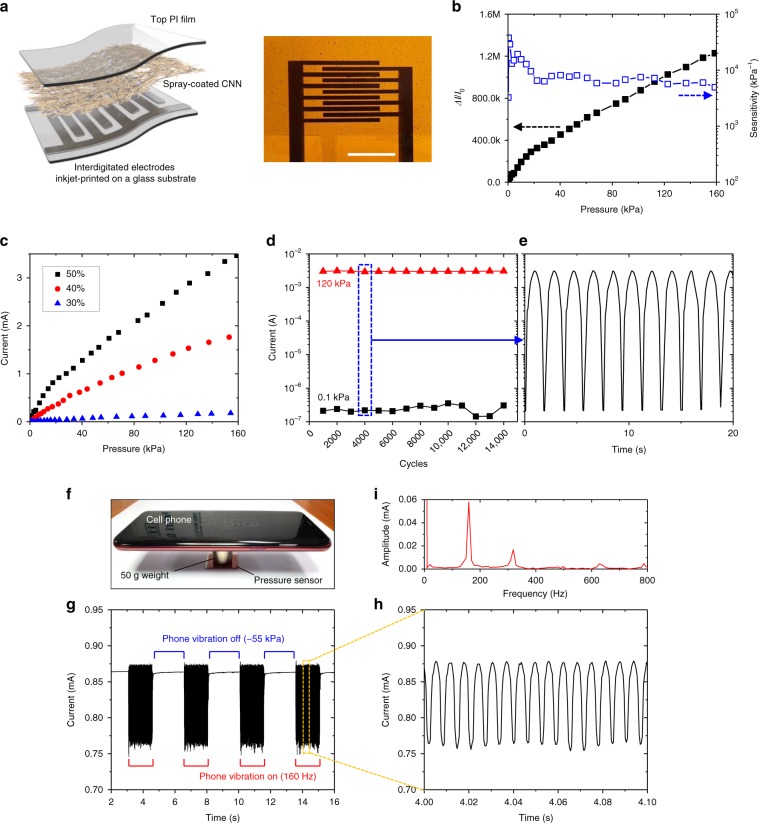
Piezoresistive characterization of CNNs. **a** Structure of CNN-based pressure sensors for piezoresistive characterization. The photograph shows the fabricated pressure sensor. Scale bar, 5 mm. **b** Pressure response and sensitivity of the pressure sensor with the 0.9-μm-thick CNN using a 50% volume ratio ink under a bias voltage of 1 V. **c** Plots of current as a function of the pressure applied onto CNN-based sensors using ink with three different volume ratios (30, 40, 50% v/v). **d** Cyclic stability of the CNN-based pressure sensor under continuous loading and unloading cycles from 0.1 kPa to 120 kPa. **e** Enlarged view of the recorded data in **d** after 4000 cycles. **f** Photograph showing the experimental setup for sensing cell phone vibration. **g** Sensing result of cell phone vibration (160 Hz) using the pressure sensor. The current was recorded at a sampling interval of 480 μs. **h** Enlarged view of the part of the current plot when the cell phone vibrated. **i** Fast Fourier transform result of the signal in **h**.

To characterize the piezoresistive performance of the CNN, we fabricated a pressure sensor by laminating a CNN-coated PI film on a glass substrate where interdigitated silver (Ag) electrodes were inkjet-printed, as illustrated in Fig. 2a (design details in Supplementary Fig. 5). Figure 2b shows a representative pressure response and the sensitivity of the pressure sensor with the 0.9-μm-thick CNN using a 50% volume ratio ink under a bias voltage of 1 V (see Supplementary Fig. 6 for a measurement method). The sensitivity is defined as *S* = δ(Δ*I*/*I*_0_)/δ*p*, where *p* denotes the applied pressure, Δ*I* denotes the current change under the applied pressure, and *I*_0_ denotes the initial current without pressure^14,33^. Our sensor showed a large sensing range (>150 kPa), maintaining its ultrahigh sensitivity (>5000 kPa^−1^) and high linearity. In the low-pressure regime (<10 kPa), the sensitivity was >10000 kPa^-1^, which far exceeded those of previously reported high-sensitivity piezoresistive pressure sensors (see Supplementary Table 1). The pressure response of the CNN was easily tuned by changing the volume ratio of the ink while maintaining high sensitivity and linearity (Fig. 2c).

The superior performance of the CNN-based pressure sensors can be attributed to the unique nanohybrid structure of the CNN (Supplementary Fig. 7). Previously reported contact-resistance-based pressure sensors generally feature that the highly conductive nanomaterials decorate cellulose microfibers or engineered microstructures. The high-density conductive materials on the microstructures form a bulky initial “conductive contact area” (CCA), i.e. contact area between electrodes and conductive nanomaterials, resulting in high *I*_0_ and consequent low *S*. On the other hand, each Te-PEDOT:PSS nanowire is encircled by the dense nanocellulose in our CNN, forming a nanostructured surface with low-density Te-PEDOT:PSS nanowires exposed on it (Fig. 1b). A small initial CCA arising from the low-density surface-exposed nanowires results in an extremely low *I*_0_ and consequent high *S*. The nanostructured surface with low-density conductive nanowires also contributes to high linearity and a large working range of the CNN. In a contact-resistance-based pressure sensor, the total electrical conductance as a function of the applied pressure can be defined as, $G_{{\mathrm{total}}}\left( P \right) = 1/R_{{\mathrm{total}}}(P) = 1/R_{{\mathrm{electrodes}}} + R_{{\mathrm{film}}} + R_{{\mathrm{contact}}}(P)$ where *R*_electrodes_ is the resistance of the finger electrodes, *R*_film_ is the resistance of the sensing film between the electrodes, and *R*_contact_(*P*) is the contact resistance between the sensing film and electrodes as a function of the applied pressure. If *R*_contact_(*P*) ≫ *R*_electrodes_ + *R*_film_, $G_{{\mathrm{total}}} \approx 1/R_{{\mathrm{contact}}}\left( P \right) = G_{{\mathrm{contact}}}(P)$, i.e. the current of the sensor is totally proportional to the *G*_contact_(*P*). When the *R*_contact_(*P*) becomes sufficiently small compared to the *R*_electrodes_ + *R*_film_, the $G_{{\mathrm{total}}} \approx 1/R_{{\mathrm{electrodes}}} + R_{{\mathrm{film}}}$ and the additional pressure no longer significantly changes the *G*_total_. According to these equations, for high linearity with a wide working range, the ${\updelta}G_{{\mathrm{contact}}}/{\updelta}P$ should be a small constant value, maintaining *R*_contact_(*P*) much higher than *R*_electrodes_ + *R*_film_. Because the bulky CCA in the microstructure-based pressure sensors results in high ${\updelta}G_{{\mathrm{contact}}}/{\updelta}P$, the *R*_contact_ rapidly decreases in a low-pressure range. Furthermore, the microstructure requires large deformation to further increase the CCA in a higher pressure range. For these reasons, the microstructure-based pressure sensors show a narrow linear region. In contrast, because of the low density of the surface-exposed Te-PEDOT:PSS nanowires in the CNN, ${\updelta}G_{{\mathrm{contact}}}/{\updelta}P$ can be maintained at a small value. In addition, the densely nanostructured surface increases the CCA uniformly and consistently, maintaining ${\updelta}G_{{\mathrm{contact}}}/{\updelta}P$ up to a higher pressure range.

Because of the dense nanohybrid structure and ultrathin design of the CNN, the current change arises mainly from the surface-exposed conductive nanowires. Consequently, much smaller deformation occurs in the sensing layer compared to the microstructure-based pressure sensors even when the high pressure is applied. As a result of the small deformation in the sensing layer, the CNN shows excellent cyclic stability and a fast response time. To investigate the cyclic stability of the CNN, we applied continuous pressure cycles on the sensor from 0.1 kPa to 120 kPa with a 120 kPa s^−1^ loading rate (Fig. 2d, e). Despite the harsh test conditions compared to those employed in previous works^33–36^, the device showed no significant change in the pressure response during and after 10,000 loading-unloading cycles, maintaining its low *I*_0_ and high sensitivity. We further measured the cyclic response of the sensor under a higher pressure condition by applying 1000 cycles each for 180 kPa, 500 kPa and 1 MPa (Supplementary Fig. 8). The sensor showed the great cyclic response corresponding to each pressure level without significant degradation. We further demonstrated the ultrafast response time of the CNN by real-time sensing of cell phone vibration. Figure 2f shows the experimental configuration for sensing cell phone vibration. We stacked a polydimethylsiloxane (PDMS) slab, a 50 g weight, and a cell phone on the sensor, where an initial pressure of ~55 kPa was applied. Figure 2g and h presents the vibration sensing results. When the phone vibrated at 160 Hz, the current measured at 480-μs intervals showed a 160-Hz oscillation with peak values of 0.88 mA and 0.76 mA, which correspond to ~60 kPa and ~45 kPa, respectively. The fast Fourier transform of the signal shown in Fig. 2i also supports that our sensor responded well to the 160 Hz vibration without any sign of damping. This ultrafast response time was also verified by the sudden pressure decrease from 16.3 to 13.6 kPa (Supplementary Fig. 9), where a response time under 1 ms was measured.

The CNN and CNN-based pressure sensors show great mechanical durability due to their ultrathin and dense nanohybrid structure. We investigated the mechanical reliability of the pressure sensor under various deformation conditions using flexible and conformable pressure sensors that have different total thicknesses. The flexible sensor comprises two 12-μm-thick PI films coated with a CNN and electrodes, respectively. The sensor showed no performance degradation after 1000 bending cycles with a bending radius of 1 mm and maintained its performance on a round surface (Supplementary Fig. 10). The conformable sensor comprises two 1-μm-thick PI films coated with a CNN and electrodes, respectively. The ultrathin sensor showed no change in its performance when it is conformably attached to a finger of a hand replica as well as after 1000 folding cycles and crumpling (Supplementary Fig. 11). The CNN-based pressure sensor showed good humidity tolerance while it showed reversible and predictable performance change under different temperature conditions because of the temperature dependence of the PEDOT:PSS^45^ (Supplementary Fig. 12).

### Super-resolution imaging of pressure distribution

**Fig. 3:**
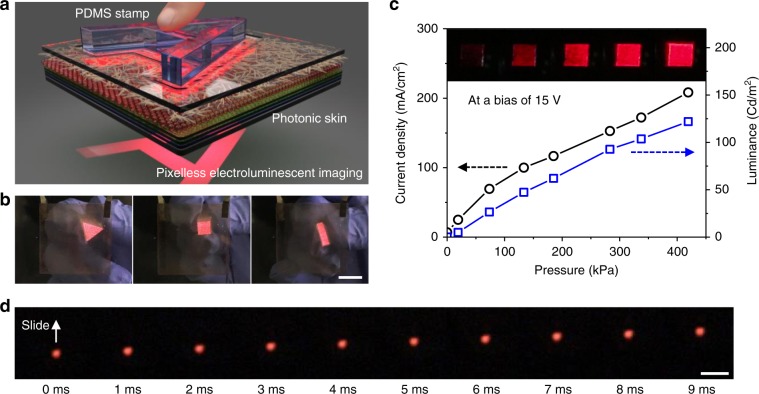
Pressure response of the photonic skin. **a** Schematic illustration of pixel-les imaging of spatially patterned constant pressure using polydimethylsiloxane (PDMS) stamps. **b** Photographs of the photonic skin visualizing the pressure applied by PDMS stamps with various shapes. Scale bar, 1 cm. **c** Current density and luminance as a function of the pressure applied with a 6 × 6 mm^2^ PDMS slab under a bias of 15 V. **d** Photographs of the pressure image captured by a high-speed camera at 1 ms intervals while a tip of a polyurethane (PU) fragment was rapidly sliding on the photonic skin, showing a fast response time of the photonic skin. Scale bar, 1 mm.

Because the engineered CNN passively controls the local current density flowing into the QLED when pressure is applied, the photonic skin understandably inherits its high sensitivity, high linearity, fast response time and great stability. Furthermore, due to the pixel-less and ultrathin design, the photonic skin displays a seamless and continuous image of the pressure distribution. To prove the reliability of the pressure map visualized by our device, we first applied spatially patterned pressure to the photonic skin on a glass substrate with PDMS stamps (Fig. 3a). Uniform light was emitted from the continuous area where the stamp was in contact with the device, visualizing the precise shape of the stamp (Fig. 3b and Supplementary Fig. 13). The pressure response of the device was investigated by measuring the current density and the luminance when a specific local pressure was applied using a 6 × 6 mm^2^ square PDMS slab (Fig. 3c and Supplementary Fig. 14). The current density and the luminance increased with high linearity up to 400 kPa at a bias of 15 V. The sensitivity, which is defined as **δ**(Δ*L*/*L*_0_)/δ*p*, where Δ*L* denotes the luminance change under the applied pressure and *L*_0_ denotes the initial luminance without pressure, was ~10 kPa^−1^ for the linear region. We investigated the response time of the photonic skin by capturing the pressure image using a high-speed camera (1000 fps) while a tip of a polyurethane (PU) fragment was rapidly sliding on the photonic skin (Supplementary Fig. 15). As shown in Fig. 3d and Supplementary Movie 1, each frame captured at 1 ms intervals showed the exact touch position of the PU tip without any trace or delay, revealing that our photonic skin has a response time of <1 ms. The fast response time of the photonic skin was also observed by an abrupt unloading process (Supplementary Fig. 16).

**Fig. 4:**
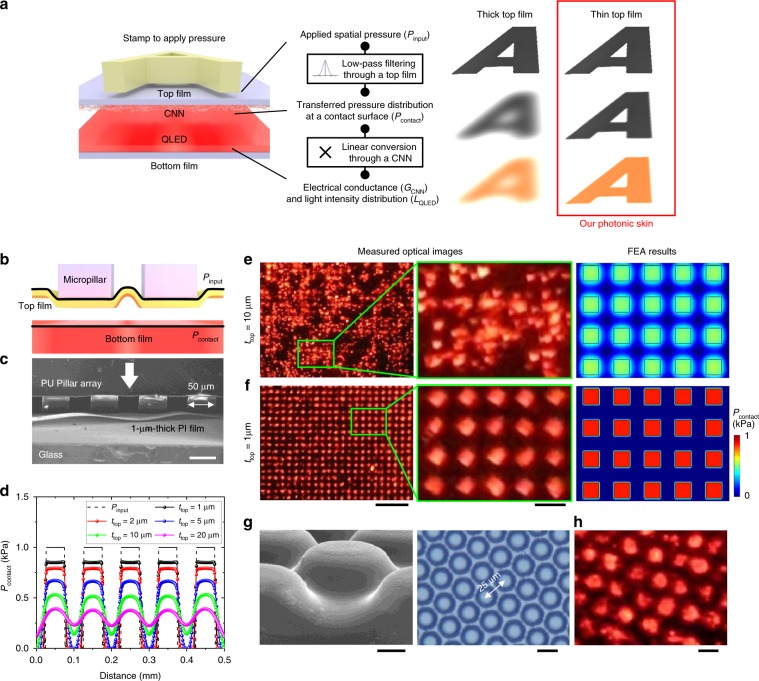
Effect of the top film thickness on the spatial resolution of the photonic skin. **a** Schematic illustration of the flow of the spatial signal when the pressure distribution (*P*_input_) is applied to the top film of the photonic skin. The *P*_input_ is low-pass filtered by the top film, forming contact pressure distribution between the CNN and the ETL (*P*_contact_). The *P*_contact_ is linearly converted into electrical conductance distribution between the CNN and the ETL (*G*_contact_) by the CNN. The light intensity distribution of the QLED (*L*_QLED_) is proportional to the *G*_contact_. **b** Schematic illustration of the situation where a micropillar array presses the top thin film. In finite element analysis (FEA), we calculated the *P*_contact_ between the top and bottom films when *P*_input_ was applied with the micropillar array. **c** SEM image where 50-μm-wide PU pillars spaced by 50 μm pressed a 1-μm-thick polyimide (PI) film on a glass substrate. Scale bar, 50 μm. **d** FEA results showing a line profile of *P*_contact_ when a pillar array with a 50 µm width and 50 µm gap (254 dpi) pressed the top film with different *t*_top_ values. **e**, **f** Optical photographs where the 254 dpi PU pillar array pressed photonic skins with *t*_top_ ~ 10 µm (**e**) and *t*_top_ ~ 1 µm (**f**). The right images present FEA results showing *P*_contact_ for the corresponding *t*_top_ values. Scale bars, 500 and 100 μm. **g** SEM and optical images of 1016 dpi PDMS hexagonal micro-bumps. Scale bars, 5 and 20 μm. **h** Optical image of the device with *t*_top_ ~ 1 µm visualizing the pressure applied with the 1016 dpi hexagonal micro-bumps. Scale bar, 20 μm.

When the spatial pressure is applied to the photonic skin, the spatial signal undergoes the following transmission process in a continuous domain: the pressure distribution applied to the top film (*P*_input_), transferred contact pressure distribution between the CNN and the ETL (*P*_contact_), electrical conductance distribution between the CNN and the ETL (*G*_contact_), and light intensity distribution of the QLED (*L*_QLED_). The flow of the spatial signal is illustrated in Fig. 4a. Because the *L*_QLED_ is proportional to the *G*_contact_ in the pixel-less QLED and the CNN linearly convert the *P*_contact_ into the *G*_contact_, if the *P*_input_ is transduced into the *P*_contact_ without distortion, the *L*_QLED_ perfectly represents the *P*_input_. Therefore, the distortion in the *P*_contact_ determines the quality of the output image and the spatial resolution of this analogue imaging. To investigate the *P*_input_/*P*_contact_ function and the spatial resolution of the photonic skin, we carried out a systematic finite element analysis (FEA) calculating the *P*_contact_ between the top and bottom films when spatially patterned *P*_input_ was applied to the top film using a micropillar array (Fig. 4b and c). As the top film conforms to the pillar, the bottom film feels as if a pillar with a larger width and rounded edges touches it, which can be seen as spatial low-pass filtering of a larger rectangular pulse. We further revealed that the passband of this low-pass filtering is inversely proportional to the top film thickness (*t*_top_) (Supplementary Note 1, 2, 3, and Supplementary Fig. 17). As a representative example, Fig. 4d shows the line profiles of *P*_contact_ in the FEA results, where a pillar array with a 50 µm width and a 50 µm gap (254 dpi) pressed the top films with different *t*_top_ values. As the *t*_top_ increased, the *P*_contact_ at the contact plane became a blurred form of *P*_input_ (see also Supplementary Note 4 and Supplementary Fig. 18). Figure 4e and f shows the experimental results where devices with *t*_top_~10μm and *t*_top_~1μm were pressed with a 254 dpi PU micropillar array. The device with *t*_top_~10μm generated a blurred and collapsed pressure image containing the large-area topography arising from the uneven pillar height and the noise due to the film roughness (Fig. 4e). On the other hand, the device with *t*_top_~1μm displayed the distinct pressure pattern applied with the 254 dpi pillar array, showing good agreement with the FEA results (Fig. 4f). This filtering effect of the thick film was also observed using higher-density micropillars, whereas the device with *t*_top_~1μm even clearly visualized the patterned pressure applied with 635 dpi micropillars (Supplementary Fig. 19) and 1016 dpi hexagonal micro-bumps (Fig. 4g, h). Importantly, the device reliably visualized both micro-texture (1016 dpi) and large-area information in pressure distribution simultaneously (Supplementary Fig. 20). For *t*_top_ = 1μm and the 1-μm-wide pillars, a spatial resolution of 4 μm (6350 dpi) was calculated by FEA, where the half maximum of *P*_contact_ was located in the middle of the two pillars (Supplementary Note 5 and Supplementary Fig. 21). These results reveal that “a pixel-less structure” and “ultrathin design” of our photonic skin are key to super-resolution analogue imaging of pressure distribution unprecedented in previously reported pressure-mapping devices (Supplementary Table 2).

**Fig. 5:**
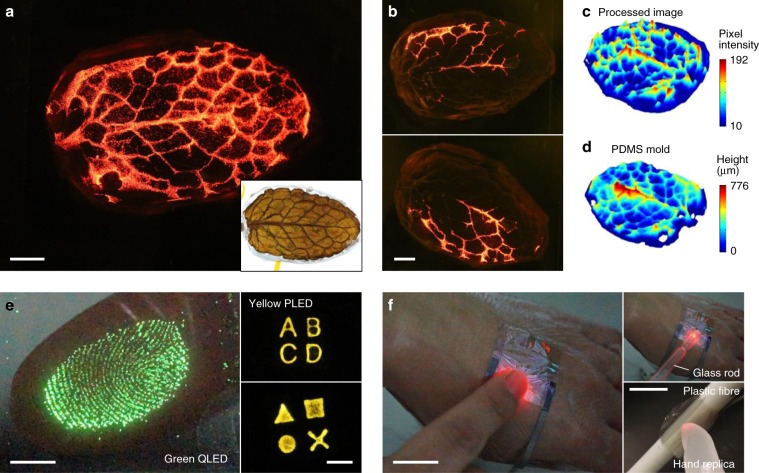
Super-resolution imaging of pressure distribution. **a**, **b** Optical photographs of the photonic skin visualizing the pressure applied with a PDMS replica of a mint leaf when the replica was fully pressed (**a**) and partially pressed (**b**). The inset in **a** shows the original mint leaf. Scale bars, 2 mm. **c** 3D plot of pixel intensity of the image in **a** after Gaussian filtering. The Gaussian filtering with *σ* = 20 was carried out to remove the noise and detailed texture information. **d** 3D surface image of the PDMS replica measured with a surface profiler. **e** Optical photographs of the photonic skins with various light-emitting devices. The left photograph shows a device with a green quantum-dot light-emitting diode (QLED) touched with a finger. The right photographs show a device with a yellow polymer light-emitting diode (PLED) touched with PDMS stamps. Scale bars, 5 mm. **f** Optical photographs of pressure imaging with freestanding devices laminated on various 3D surfaces. When a finger and a glass rod touched the photonic skin laminated on a human hand and a plastic fibre, it well visualized pressure distribution in real-time. Scale bars, 2 cm.

Notably, this super-resolution and high-linearity pressure imaging enables 3D surface mapping of an elastic object because the contact pressure between the device and the elastic body is proportional to the compressive strain normal to the contact plane. We mapped the 3D surface of a mint leaf by capturing and processing the image generated on the device where a PDMS replica of the mint leaf was fully pressed (Supplementary Fig. 22). The device distinctly displayed the surface morphology of the compressed part of the leaf replica, including major veins and a more detailed skeleton (Fig. 5a, b, and Supplementary Movie 2). The higher light intensity corresponds to the higher height of the PDMS replica. After Gaussian filtering of the captured image to eliminate noise and micro-texture information (Supplementary Fig. 23), a 3D pixel intensity map was generated (Fig. 5c). Compared to the 3D surface image of the PDMS replica measured using a surface profiler in Fig. 5d, the generated image well represented the height map of the PDMS replica. The distortion mainly arose from the incomplete compression and the unevenly applied pressure.

Moreover, our device generates high-quality and high-contrast images of valuable micro-textures such as a fingerprint (Fig. 1d and Fig. 5e). A clear fingerprint pattern appeared with the light intensity corresponding to the touch force when the device was touched. Photonic skins with a green QLED and a yellow polymer light-emitting diode (PLED) were also demonstrated, showing the universality of our approach (Fig. 5e). The most compelling advantage of our pressure-sensitive photonic skin is that it can operate while conformably laminated on 3D surfaces, just like “real skin.” The operation of the ultraflexible devices laminated on human skin and a plastic fibre is shown in Fig. 5f. When a finger and a glass rod touched the photonic skins attached onto the hand and the plastic fibre, they showed touch information in real-time (Supplementary Movie 3). The device also visualized the pressure at valleys of wrinkles generated around the location of touch, which could be a good example of our device visualizing even unintended force, implying its high sensitivity and reliability.

Our high-fidelity analogue imaging with unprecedented response time (<1 ms) can be rapidly digitized by digital imaging technologies (e.g. high-resolution charge-coupled devices (CCDs)) in real-time, which could be challenging in high-resolution sensor arrays due to the complicated data acquisition process and scanning time of a large number of pixels. Once the light intensity distribution is captured, it can be transformed into actual pressure data based on a lookup table or pre-calibrated data. The luminance as a function of the applied pressure in Fig. 3c is a great example of such lookup tables because the luminance is one of the absolute measurements of the light intensity. Alternatively, the pixel intensity from a CCD as a function of the applied pressure could be another simple option. To demonstrate the whole data transformation process, we transformed the digitized electroluminescent image into actual pressure distribution using a lookup table for pixel intensity (Supplementary Fig. 24). We measured the mean pixel intensity of the electroluminescent image captured by a CCD for a specific uniform pressure applied with a 6 × 6 mm^2^ square PDMS slab. By using this lookup table, the pressure distribution was directly calculated from the photograph captured by a CCD when the photonic skin was pressed with a B-shaped stamp. This simple demonstration highlights the feasibility of the data transformation from the electroluminescent image of our photonic skin to the actual pressure distribution.

### Application to real-time smart touch interfaces

**Fig. 6:**
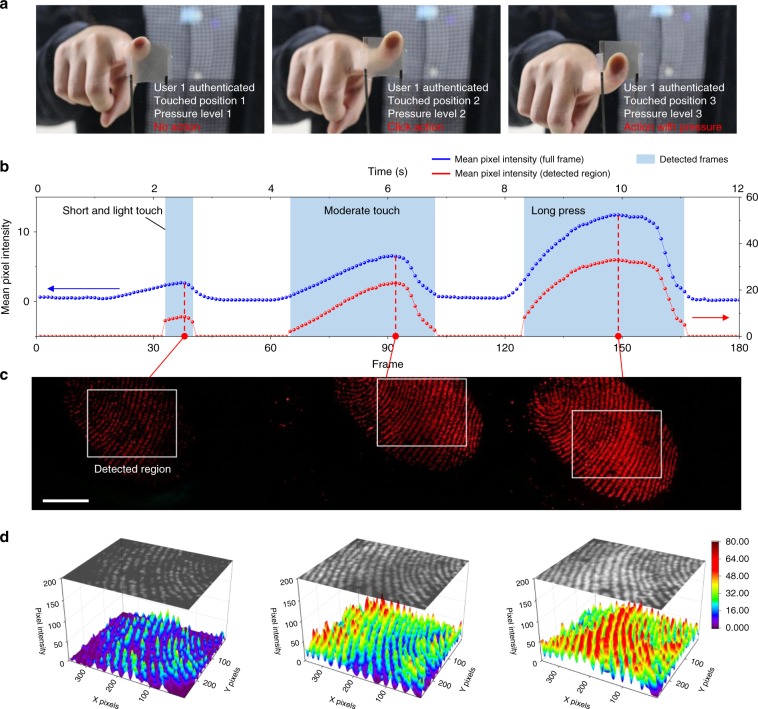
Application to real-time smart touch interfaces. **a** Conceptual illustration of smart touch interfaces that can identify the user and exact location of touch using the photonic skin. **b** Mean pixel intensities (MPIs) of the images captured by a charge-coupled device (CCD) at 15 fps where the device was touched with a finger three times with different positions, forces and durations (blue dots: MPIs of full-size images, red dots: MPIs of the regions detected as a fingerprint of the user). **c** Three frames containing the detected regions when the touch force reached the maximum value during each touch. White rectangles denote the regions detected as a fingerprint of the user. Scale bar, 5 mm. **d** Pixel intensity maps for the Gaussian-filtered images (*σ* = 3) of the detected regions in **c**.

We further demonstrated novel real-time high-information-density human-machine interfaces using our photonic skin, namely, “smart touch interfaces.” Fig. 6a illustrates the concept of the smart touch interface, which is capable of identifying the user in addition to sensing the touch force and location in real-time. From this type of touch interface, a variety of new machine actions could be generated. For a proof of concept, the device was touched with a finger three times with different positions, forces and durations, and the generated images were recorded by a CCD at 15 frames per second. These images were transferred to a computer that performed fingerprint recognition by a simple template matching algorithm and examined the exact touch location and relative force in real-time (Supplementary Movie 4). The mean pixel intensities (MPIs) for the full image and the region detected as a fingerprint of the user are plotted as a function of the frame number in Fig. 6b. Although the MPIs for both the full area and detected region well represented the relative touch force, the MPI of the detected region was more sensitive as a result of excluding the untouched area. Figure 6c shows the three frames containing the detected regions when the touch force reached the maximum value during each touch. These images show that our smart touch interface can calculate the exact location of the touch from the micro-texture information even if there is an unintended touch or the size of the contact area varies. The 3D plots of the pixel intensities in the detected regions in Fig. 6d further provide the detailed fingerprint morphology and the associated pressure profile. This demonstration highlights the feasibility of fast and super-resolution pressure imaging with our device for demanding applications.

## Discussion

We introduced the first fully conformable and imperceptible electronics capable of imaging pressure distribution with high spatial and temporal fidelity without the need for pixel structures. The proposed pixel-less device offers numerous unique advantages that previous works employing patterned active pixels cannot realize. In terms of imaging performance, owing to the homogeneous and ultrathin design, our device emits light from a continuous surface visualizing the precise shape and magnitude of the applied pressure. The ultrahigh sensitivity of the CNN enables high-light-intensity and high-contrast representation for a practical pressure regime (<1 MPa). High linearity and fast response time (<1 ms) enable novel practical applications such as 3D surface mapping and smart touch interfaces. In terms of device design, our device has an ultrathin and transparent structure, forming an imperceptible interface with human skin. Furthermore, the simple and low-cost fabrication procedure including a novel water-based method for the CNN is even applicable for large-area devices, showing its feasibility (Supplementary Fig. 25).

The results here provide a new experimental and theoretical framework for revealing an accurate stimulus profile by exploiting the conformability of an ultrathin film. Theoretical postulation and an FEA reveal that the top film acts as a spatial low-pass filter for the applied force and that the thickness of the film determines the spatial resolution. For a 1-μm-thick top film, a resolution of >6000 dpi is calculated by the FEA, and the experimental results show a clear pressure map generated by micropillars of >1000 dpi. Obviously, both values from the experiments and FEA are much higher than those of previous sensor-array-based pressure-mapping devices that are generally under 100 dpi (Supplementary Table 2). Although pressure-imaging devices using piezoelectric nanowire LEDs have achieved high pixel resolution (~6000 dpi)^29–31^, their extremely low sensitivity, low fill factor and lack of conformability hinder their practical applications. For example, due to the low sensitivity (a few tens of GPa^−1^) of such devices, they cannot detect the practical pressure distribution applied by soft bodies such as a fingerprint. In contrast, because of the high sensitivity of our photonic skin, it can clearly map a pressure profile arising from a microscale surface morphology of soft bodies with low Young’s modulus. Furthermore, a wide working range with great linearity makes the resulting electroluminescent image to be much closer to the accurate pressure distribution. Future work would include theoretical analyses of pressure transferring through a thin film, practical improvements in the device performance such as encapsulation of the device, and integration with pre-established and emerging technologies such as microfluidics and deformable displays. These directions may satisfy the demand for fully understanding a spatial stimulus in future applications such as display-integrated advanced user interfaces, implantable bio-imaging systems and nervous systems in soft robotics.

## Methods

### Fabrication of CNNs and pressure sensors

Te-PEDOT:PSS nanowires were synthesized based on previously reported methods^46^. First, 1 g of L-ascorbic acid was dissolved in 45 mL of deionized water. Then, 1 mL of PEDOT:PSS (Clevios PH1000) solution filtered through a PVDF syringe filter was added to this solution. Subsequently, 0.07 g of Na_2_TeO_3_ was added to the mixture under stirring. The temperature of the mixture was then increased to 90 °C and maintained for 24 h. Te-PEDOT nanorods were collected by centrifugation of the reaction mixture and suspended in 5 mL of deionized water. The final dispersion had a concentration of 78 mg mL^−1^. The fabricated Te-PEDOT:PSS nanowires had a length of 1 μm and a diameter of ~10 nm.

A homogeneously nanofibrillated cellulose suspension was fabricated based on our previous work^47^. A 1 wt% aqueous dispersion of cellulose powder (Aldrich) was chemically treated by potassium hydroxide (2 wt%) and sodium chloride (2 wt%) at 60 °C for 2 h to remove lignin and hemicelluloses. After the chemical treatment, the samples were rinsed with DI water until the residues were neutralized. This water slurry was passed 30 times through a high-pressure homogenizer under 1500 bar (Nano Disperser, Suflux) and finally dispersed in DI water at a concentration of 90 mg mL^−1^. The cellulose was nanofibrillated with a diameter of 10–20 nm and a length of 1–2 μm.

Aqueous dispersions of Te-PEDOT nanowires and cellulose nanofibres were mixed with various volume ratios (30:70, 40:60, 50:50). CNNs were deposited on 50-μm-thick PI films held at 100 °C by spray-coating the mixtures using a commercial airbrush at a distance of ~10 cm. Multiple passes of airbrushing were performed until the desired thickness was reached. The coated substrates were dried on a hotplate at 100 °C for 30 min to remove residual solvent. Interdigitated Ag electrodes were deposited onto a glass substrate by inkjet-printing of nanoparticle-type Ag ink (DGP 40LT-15C, ANP). The total size of the finger electrodes was 6 × 6 mm^2^, and the width and gap of the electrodes were 400 μm and 200 μm, respectively (see Supplementary Fig. 5). The PI film was laminated onto the glass substrate using double-sided adhesive tape.

### Fabrication of pressure-sensitive photonic skin

A colourless PI varnish was gently provided by KOLON Corporation, Korea. A 1-μm-thick colourless PI film (bottom PI) was coated on a glass substrate at 5000 r.p.m. and then cured at 150 °C for 10 min and 200 °C for 2 h on a hotplate. Then, an ITO electrode (150 nm) was deposited by sputtering onto the colourless PI film. The ITO-coated colourless PI film on carrier glass was treated with O_2_-plasma for 30 s. PEDOT:PSS (Batron P VP AI 4083, filtered through a 0.45 μm PES filter) as a hole injection layer (HIL) was spin-coated on the ITO anode at 2000 r.p.m. for 30 s, followed by annealing at 150 °C for 10 min. Poly(9-vinylcarbazole) (PVK, in chlorobenzene, 10 mg mL^−1^) as a hole transport layer (HTL) was spin-coated on the HIL at 4000 r.p.m. for 30 s, followed by annealing at 120 °C for 10 min. An emissive layer of colloidal CdSe/ZnS quantum dots (QDs, in toluene, 10 mg mL^−1^, Mesolight) was spin-coated on the HTL at 4000 r.p.m. for 30 s and annealed at 60 °C for 20 min. A ZnO nanoparticle dispersion (diluted in 1-butanol, 5 wt%, Avantama AG) was spin-coated on the QD layer at 4000 r.p.m. for 30 s and annealed at 60 °C for 20 min. The top colourless PI film (1-μm-thick) was coated on the other glass substrate at 5000 r.p.m. and then cured at 150 °C for 10 min and 200 °C for 2 h on a hotplate, and AgNWs (0.5 wt%, NANOPYXIS) were spin-coated as a common cathode and annealed at 120 °C for 10 min. The CNN (50% v/v) was spray-coated onto the AgNW cathode, followed by baking at 100 °C for 30 min. The top PI film on the supporting glass was scribed on all four edges before lamination and then laminated on the bottom PI film on the bottom glass using 20-μm-thick double-sided adhesive tape. The top glass was detached from the top PI film, leaving the top PI film attached onto the bottom film. Subsequently, the whole device was cut on all four edges and was mechanically detached from the bottom supporting glass.

### Piezoresistive characterization of CNNs

Electrical characterization of the pressure sensors was carried out using a Keithley 2400 SourceMeter and an Agilent 4155c semiconductor parameter analyser under a bias voltage of 1 V. Static loads and continuous loading and unloading cycles were applied with an automatic force test stand (ASM-1000, a length resolution of 10 μm, Digitech) with a computer controller. The sheet resistances and transmittances of the CNNs were measured using an ohm meter with a four-point probe (FPP-RS 8, DASOL ENG) and a spectrometer (DH-2000-BAL, Ocean optics), respectively.

### Pressure imaging with the photonic skin

The pressure responses of the photonic skin were basically studied using devices on supporting glass substrates. The luminance of the photonic skin was measured using a colour and luminance meter (CS-200, Konica Minolta) while applying loads with a 6 × 6 mm^2^ square PDMS slab attached to a portable digital force gauge (FGN-50B, SHIMPO).

PDMS stamps with various shapes were fabricated by replica moulding processes using 3D-printed prototype models. A PDMS precursor (Sylgard 184, Dow Corning) was poured on the 3D-printed models and cured at 100 °C for 2 h. The PDMS master moulds were detached from the prototypes. Monolayers of 1 H,1 H,2 H,2H-perfluorooctyltrichlorosilane (FOTS) were vapour-deposited on the O_2_-plasma-treated surfaces of the PDMS master moulds for anti-stiction. A PDMS precursor was then poured on the master moulds and cured at 100 °C for 2 h. After being detached from the mater moulds, PDMS stamps with various shapes were obtained. A PDMS replica of a mint leaf was fabricated using the same process except that the leaf replaced the 3D-printed prototypes.

PU micropillar arrays for a spatial resolution study were fabricated by similar replica moulding processes using micro-patterned silicon substrates as master moulds. In this study, 20-μm-deep square holes with various sizes were patterned on the silicon substrates by photolithography. Next, a 300-nm-thick aluminium film was sputtered on the silicon substrate. The aluminium film was patterned by inductively coupled plasma (ICP) etching through a patterned photoresist. After removal of the remaining photoresist, the silicon substrate was etched using deep reactive-ion etching (DRIE) through the aluminium mask to obtain 20-μm-deep square holes. Finally, the remaining aluminium mask was removed using ICP etching to obtain micro-patterned silicon master moulds.

A PDMS micro-bump array was fabricated by microsphere lithography. A closely packed, hexagonal monolayer of polystyrene (PS) microspheres with a diameter of 25 μm (Aldrich) was first formed on a PDMS substrate by a previously reported rubbing method^48^. Another flat PDMS substrate was used for rubbing the microspheres. After the rubbing process, the PDMS substrate was etched by reactive-ion etching (RIE) process for 80 min using the PS microsphere monolayer as an etching mask. Oxygen (O_2_) and tetrafluoromethane (CF_4_) with a ratio of 1:5 were used for process gases. After removing the remaining PS microspheres from the PDMS substrate using adhesive tape, a hexagonally aligned PDMS micro-bump array was obtained.

### SEM and TEM observation

The surface and cross-sectional morphologies of the CNN and the photonic skin were examined using field emission scanning electron microscopy (SEM, FEI Sirion 400, SGC equipment) and transmission electron microscopy (TEM, Tecnai G^2^ F30 S-TWIN, FEI). The samples were sliced using a focused ion beam (FIB) system (Dual Beam FIB Nova 200, FEI)

## Supplementary information

Supplementary Information

Description of Additional Supplementary Information

Supplementary Movie 1

Supplementary Movie 2

Supplementary Movie 3

Supplementary Movie 4

## Data Availability

The authors declare that all data supporting the findings of this study are available within the article and its Supplementary Information files or from the corresponding author upon reasonable request.

## References

[1] Lee H (2016). A graphene-based electrochemical device with thermoresponsive microneedles for diabetes monitoring and therapy. Nat. Nanotechnol..

[2] Yokota T (2016). Ultraflexible organic photonic skin. Sci. Adv..

[3] Kang S-K (2016). Bioresorbable silicon electronic sensors for the brain. Nature.

[4] Kim Y (2018). A bioinspired flexible organic artificial afferent nerve. Science.

[5] Jung S (2014). Reverse-Micelle-induced porous pressure-sensitive rubber for wearable human-machine interfaces. Adv. Mater..

[6] Cañón Bermúdez GS (2018). Magnetosensitive e-skins with directional perception for augmented reality. Sci. Adv..

[7] Wang S (2018). Skin electronics from scalable fabrication of an intrinsically stretchable transistor array. Nature.

[8] Hua Q (2018). Skin-inspired highly stretchable and conformable matrix networks for multifunctional sensing. Nat. Commun..

[9] Kaltenbrunner M (2013). An ultra-lightweight design for imperceptible plastic electronics. Nature.

[10] Park S (2018). Self-powered ultra-flexible electronics via nano-grating-patterned organic photovoltaics. Nature.

[11] Mannsfeld SCB (2010). Highly sensitive flexible pressure sensors with microstructured rubber dielectric layers. Nat. Mater..

[12] Fan F-R (2012). Transparent triboelectric nanogenerators and self-powered pressure sensors based on micropatterned plastic films. Nano Lett..

[13] Pan L (2014). An ultra-sensitive resistive pressure sensor based on hollow-sphere microstructure induced elasticity in conducting polymer film. Nat. Commun..

[14] Bae GY (2016). Linearly and highly pressure-sensitive electronic skin based on a bioinspired hierarchical structural array. Adv. Mater..

[15] Amjadi M, Yoon YJ, Park I (2015). Ultra-stretchable and skin-mountable strain sensors using carbon nanotubes-Ecoflex nanocomposites. Nanotechnology.

[16] Cai G (2017). Extremely stretchable strain sensors based on conductive self-healing dynamic cross-links hydrogels for human-motion detection. Adv. Sci..

[17] Trung TQ, Ramasundaram S, Hwang B-U, Lee N-E (2016). An all-elastomeric transparent and stretchable temperature sensor for body-attachable wearable electronics. Adv. Mater..

[18] Gao W (2016). Fully integrated wearable sensor arrays for multiplexed in situ perspiration analysis. Nature.

[19] Kim J (2014). Stretchable silicon nanoribbon electronics for skin prosthesis. Nat. Commun..

[20] Gerratt AP, Michaud HO, Lacour SP (2015). Elastomeric electronic skin for prosthetic tactile sensation. Adv. Funct. Mater..

[21] Kim H-J, Sim K, Thukral A, Yu C (2017). Rubbery electronics and sensors from intrinsically stretchable elastomeric composites of semiconductors and conductors. Sci. Adv..

[22] Byun J (2018). Electronic skins for soft, compact, reversible assembly of wirelessly activated fully soft robots. Sci. Robot..

[23] Kamaya M, Kawakubo M (2011). A procedure for determining the true stress-strain curve over a large range of strains using digital image correlation and finite element analysis. Mech. Mater..

[24] Takei K (2010). Nanowire active-matrix circuitry for low-voltage macroscale artificial skin. Nat. Mater..

[25] Lin L (2013). Triboelectric active sensor array for self-powered static and dynamic pressure detection and tactile imaging. ACS Nano.

[26] Wang X (2016). Self-powered high-resolution and pressure-sensitive triboelectric sensor matrix for real-time tactile mapping. Adv. Mater..

[27] Wang X (2017). Full dynamic-range pressure sensor matrix based on optical and electrical dual-mode sensing. Adv. Mater..

[28] Wang C (2013). User-interactive electronic skin for instantaneous pressure visualization. Nat. Mater..

[29] Pan C (2013). High-resolution electroluminescent imaging of pressure distribution using a piezoelectric nanowire LED array. Nat. Photonics.

[30] Peng M (2015). High-resolution dynamic pressure sensor array based on piezo-phototronic effect tuned photoluminescence imaging. ACS Nano.

[31] Bao R (2016). CdS nanorods/organic hybrid LED array and the piezo-phototronic effect of the device for pressure mapping. Nanoscale.

[32] Wang X (2015). Dynamic pressure mapping of personalized handwriting by a flexible sensor matrix based on the mechanoluminescence process. Adv. Mater..

[33] Gong S (2014). A wearable and highly sensitive pressure sensor with ultrathin gold nanowires. Nat. Commun..

[34] Zhan Z (2017). Paper/carbon nanotube-based wearable pressure sensor for physiological signal acquisition and soft robotic skin. ACS Appl. Mater. Interfaces.

[35] Tao L-Q (2017). Graphene-paper pressure sensor for detecting human motions. ACS Nano.

[36] Wei Y, Chen S, Lin Y, Yuan X, Liu L (2016). Silver nanowires coated on cotton for flexible pressure sensors. J. Mater. Chem. C..

[37] Chen L, Chen G, Lu L (2007). Piezoresistive behavior study on finger-sensing silicone rubber/graphite nanosheet nanocomposites. Adv. Funct. Mater..

[38] Xu X (2017). Copper nanowire-based aerogel with tunable pore structure and its application as flexible pressure sensor. ACS Appl. Mater. Interfaces.

[39] Yao H-B (2013). A flexible and highly pressure-sensitive graphene-polyurethane sponge based on fractured microstructure design. Adv. Mater..

[40] Lv L, Zhang P, Xu T, Qu L (2017). Ultrasensitive pressure sensor based on an ultralight sparkling graphene block. ACS Appl. Mater. Interfaces.

[41] Hou C, Wang H, Zhang Q, Li Y, Zhu M (2014). Highly conductive, flexible, and compressible all-graphene passive electronic skin for sensing human touch. Adv. Mater..

[42] Choong C-L (2014). Highly stretchable resistive pressure sensors using a conductive elastomeric composite on a micropyramid array. Adv. Mater..

[43] Park J (2014). Giant tunneling piezoresistance of composite elastomers with interlocked microdome arrays for ultrasensitive and multimodal electronic skins. ACS Nano.

[44] Jian M (2017). Flexible and highly sensitive pressure sensors based on bionic hierarchical structures. Adv. Funct. Mater..

[45] Nardes AM, Kemerink M, Janssen RAJ (2007). Anisotropic hopping conduction in spin-coated PEDOT:PSS thin films. Phys. Rev. B.

[46] Bae EJ, Kang Y-H, Jang K-S, Cho S (2016). Enhancement of thermoelectric properties of PEDOT:PSS and Tellurium-PEDOT:PSS hybrid composites by simple chemical treatment. Sci. Rep..

[47] Park N-M (2017). Electroluminescent nanocellulose paper. Mater. Lett..

[48] Park C (2014). Quick, large-area assembly of a single-crystal monolayer of spherical particles by unidirectional rubbing. Adv. Mater..

