# Supplementary material for: Ultraflexible and transparent electroluminescent skin for real-time and super-resolution imaging of pressure distribution
Source: Nat Commun. 2020 Jan 31;11:663. doi: 10.1038/s41467-020-14485-9 (PMC6994701; doi:10.1038/s41467-020-14485-9)
Supplement: Supplementary file 1 — Supplementary Information [file 41467_2020_14485_MOESM1_ESM.pdf]

Supplementary Information

Ultraflexible and transparent electroluminescent skin for real-time and super-resolution imaging of pressure distribution

Lee *et al.*

Supplementary Figures

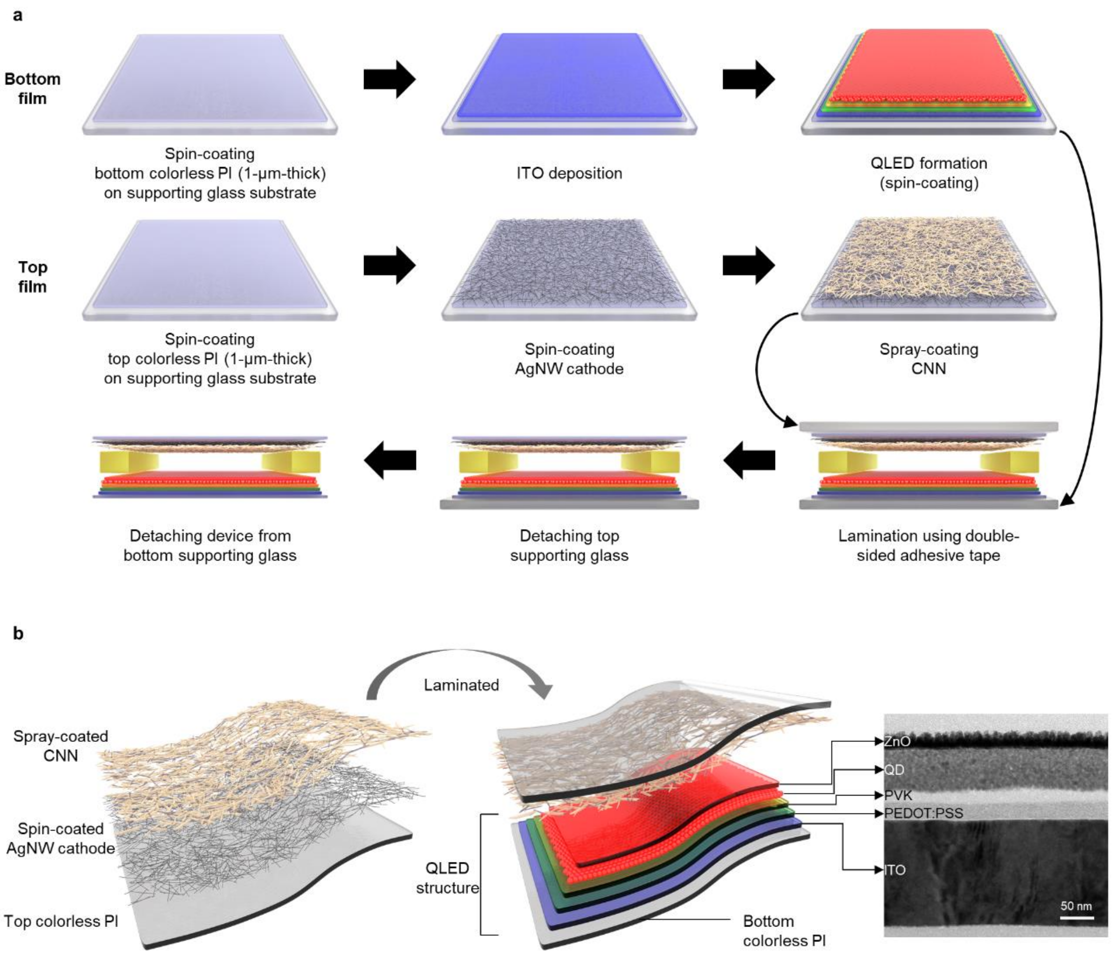

Supplementary Figure 1 | Fabrication process for the pressure-sensitive photonic skin. a and **b**, Schematic illustration of the fabrication process (**a**) and the detailed structure (**b**) of the pressure-sensitive photonic skin. The right inset in **b** is a TEM image of the QLED structure.

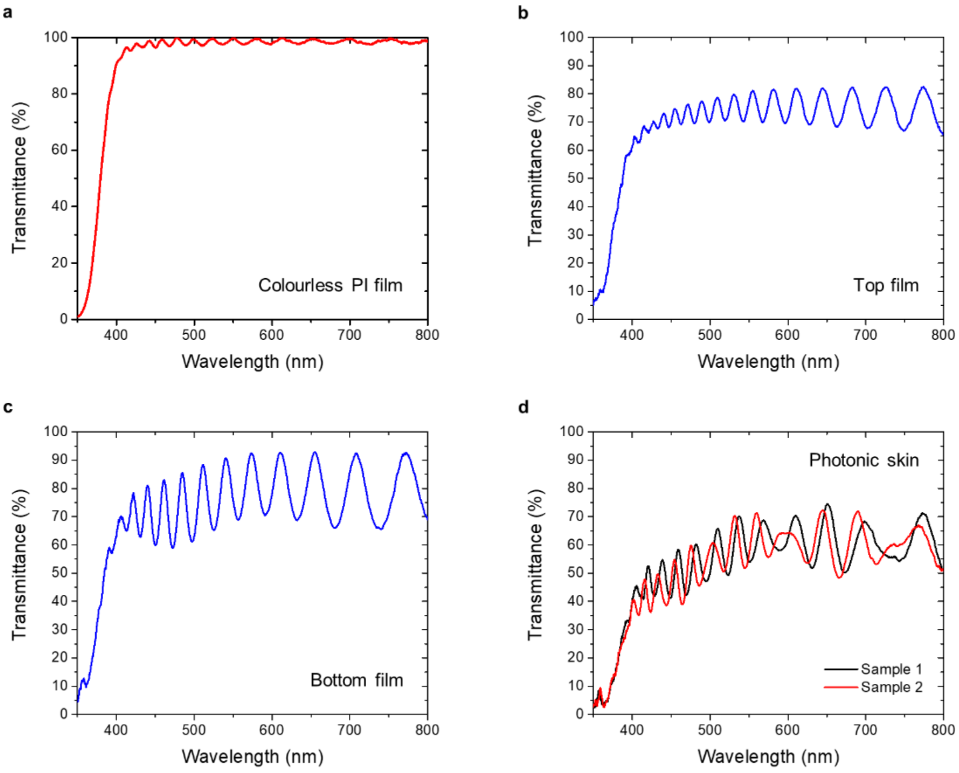

Supplementary Figure 2 | Transmittance of the photonic skin. a, **b**, **c**, and **d**, Transmittances of the colourless PI film (**a**), top sensing film (**b**), the bottom light-emitting film (**c**) and the photonic skin (**d**).

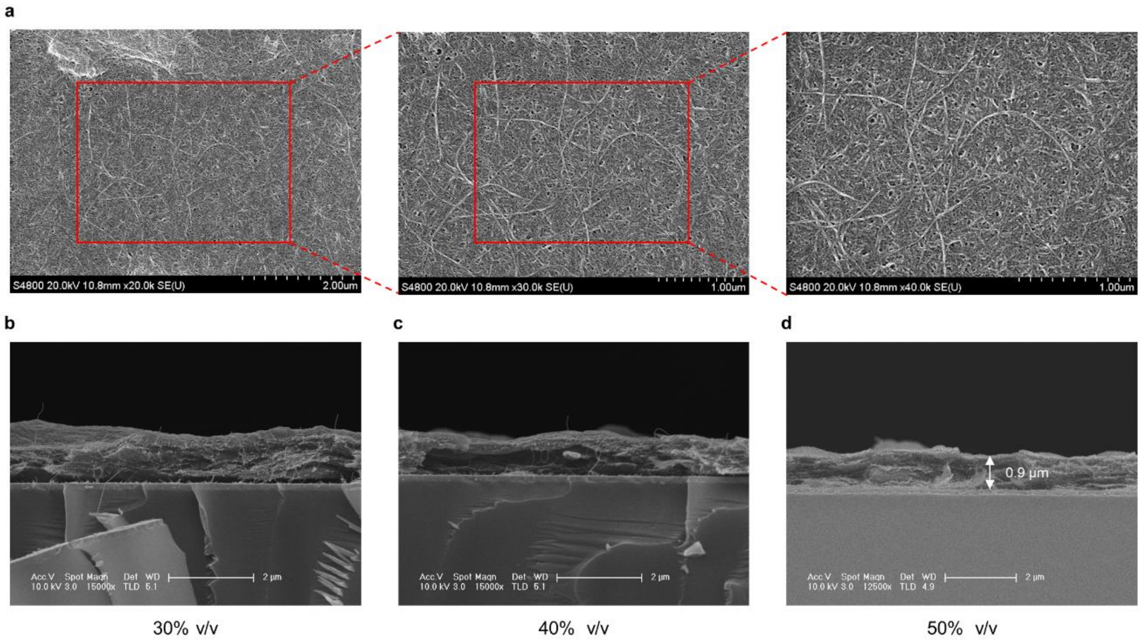

Supplementary Figure 3 | Surface and cross-sectional morphologies of the CNNs. a, SEM images of the surface of the CNN. **b**, **c** and **d**, Cross-sectional SEM images of CNNs prepared using 6 mL of 30% (**b**), 40% (**c**) and 50% v/v ink (**d**).

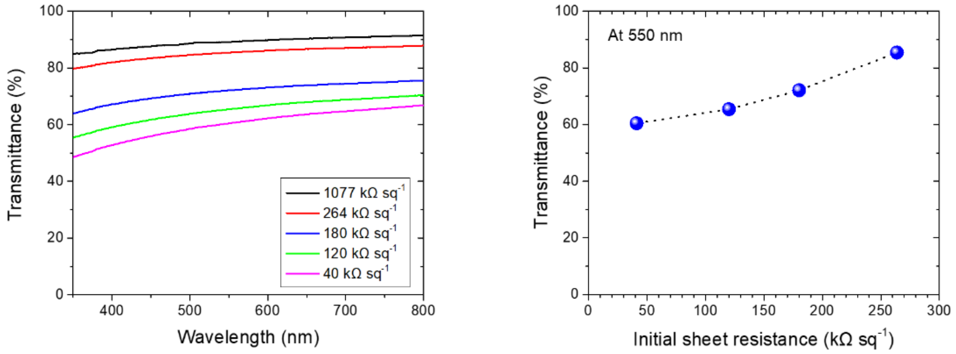

Supplementary Figure 4 | Transmittance of the CNN as a function of sheet resistance.

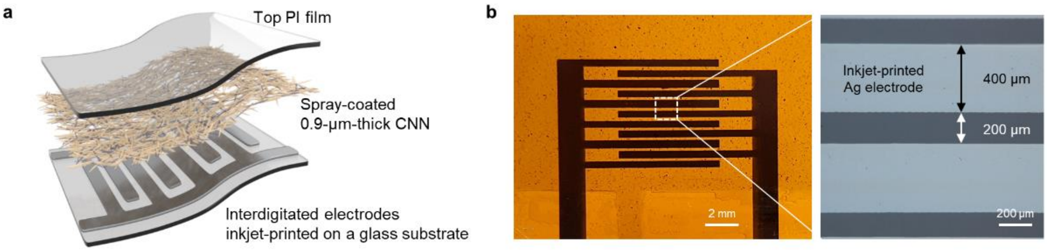

Supplementary Figure 5 | Structure of the CNN-based pressure sensors. a, Schematic illustration of the structure of the CNN-based pressure sensor. **b**, Optical images of the pressure sensors and the inkjet-printed interdigitated electrodes. The thickness of the PI film was 50 µm, the total size of the interdigitated electrodes was 6 × 6 mm^2, and the width and gap of the electrodes were 400 µm and 200 µm, respectively.

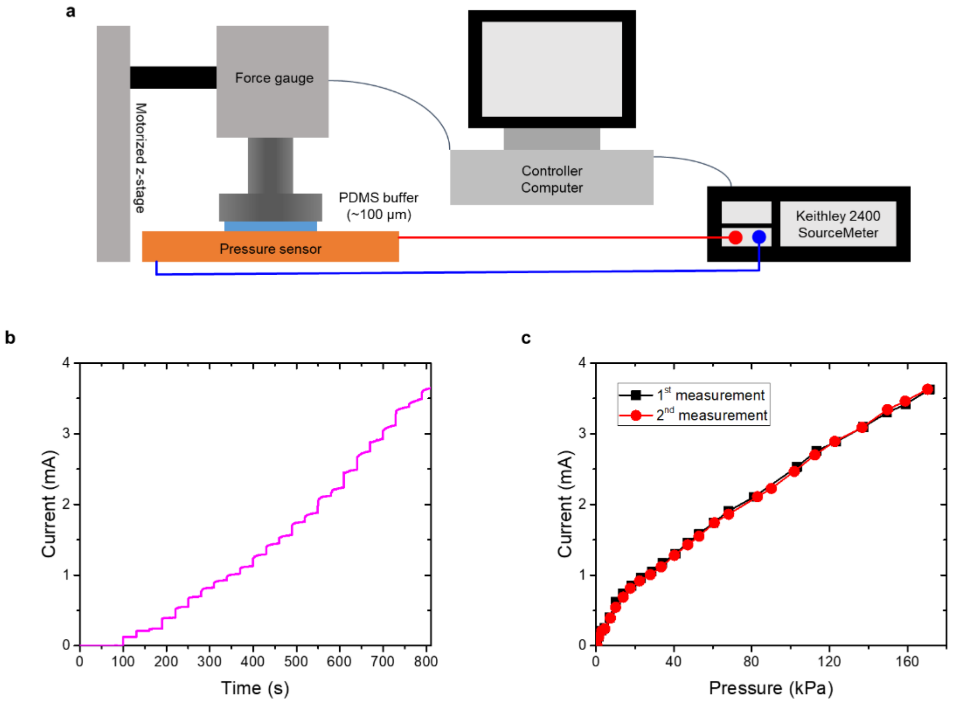

Supplementary Figure 6 | Piezoresistive characterization. a, Schematic illustration of the experimental setup for piezoresistive characterization of the CNN-based pressure sensors. A 100-µm-thick PDMS buffer was placed between the pressure sensor and the force gauge for uniform pressure application on a precise area. The step-by-step pressures were applied by controlling the motorized z-stage. **b**, Time-resolved raw data of the measured current while applying step-by-step pressure. **c**, Reliability and stability of the pressure response of the CNN-based pressure sensor. There is no significant difference between the two repeated measurements.

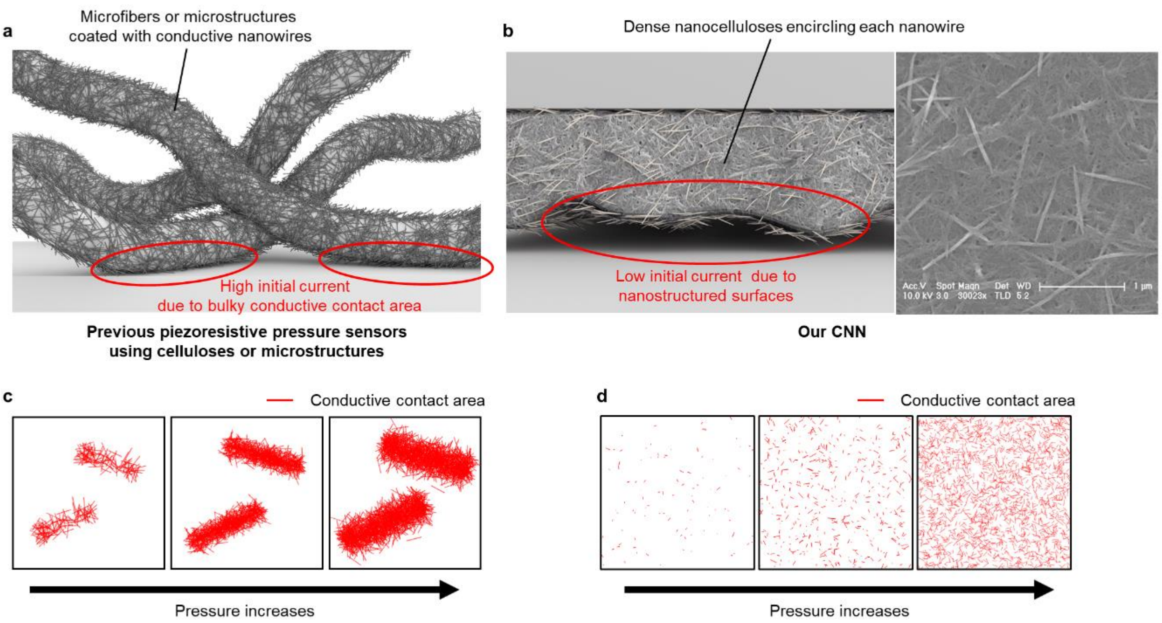

Supplementary Figure 7 | Principle of ultrahigh sensitivity and linearity. **a** and **b**, Schematic illustration of the mechanism of previous piezoresistive pressure sensors using cellulose or microstructures (**a**) and our CNN (**b**). The previous piezoresistive pressure sensors, where the microfibres or microstructures such as tissue paper or a sponge are coated with conductive nanowires, show a bulky "conductive contact area", resulting in a high initial current. Here, the conductive contact area (CCA) denotes the contact area between the conductive nanowires and the electrodes. In contrast, our CNN features that the cellulose nanofibers encircle each nanowire, forming a dense nanostructure as shown in the right SEM image in **b**. This nanostructured surface results in a small CCA and consequently an extremely low off-current. A small pressure could initiate a drastic increase in the CCA. **c** and **d**, Schematic illustration of the CCA change in previous sensors (**c**) and the CNN (**d**) when pressure is applied. The CCA in the previous sensors expands mainly from the initial contact area, requiring large deformation of the microstructures. On the other hand, the CCA in our CNN uniformly increases in the whole area, showing increases in both the number of contact nanowires and the contact area of each nanowire. These unique properties give rise to the ultrahigh sensitivity and linearity over a wide working range.

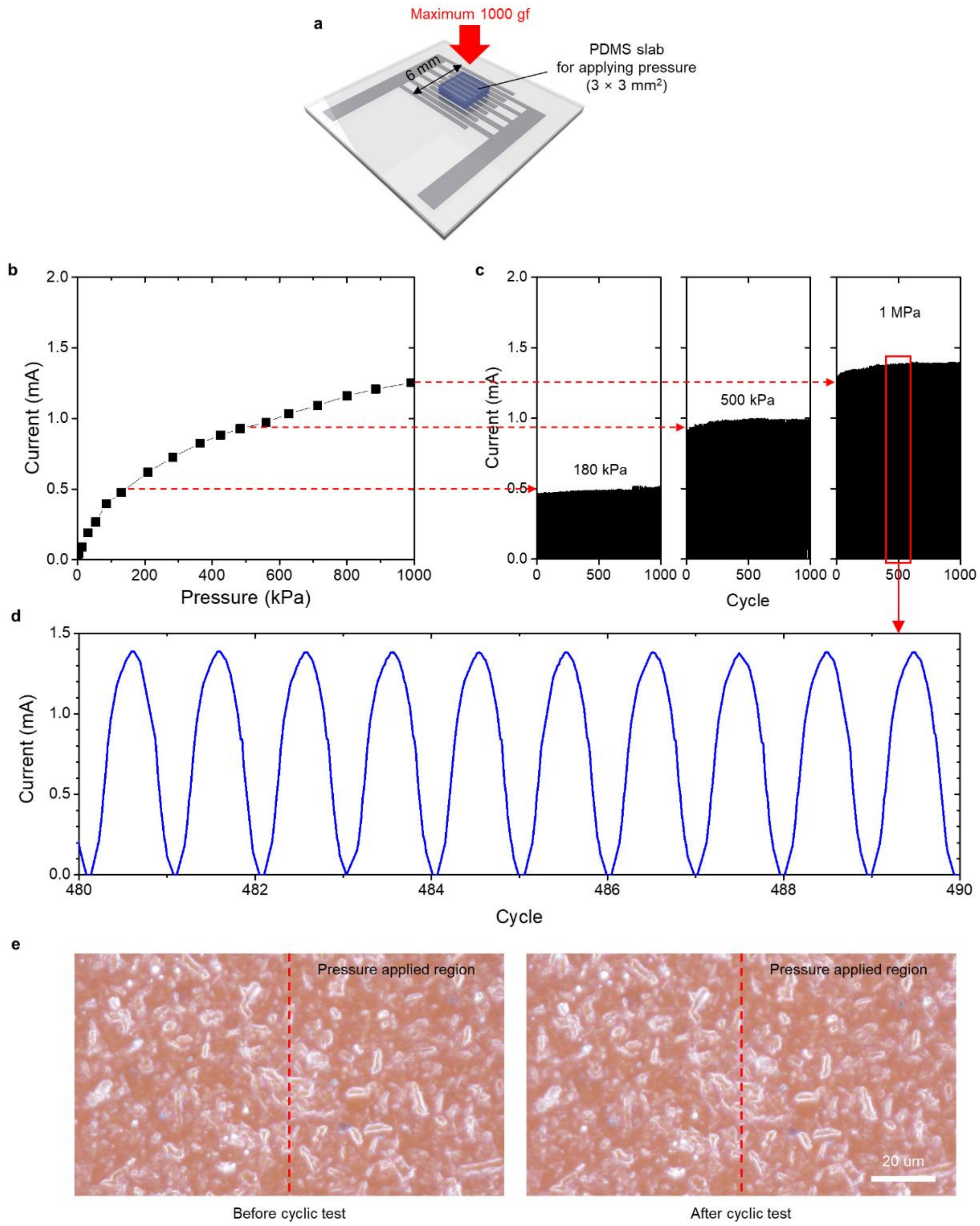

Supplementary Figure 8 | High pressure reliability. a, Schematic illustration of an experimental setup for a high pressure reliability test. **b**, Pressure response of the CNN-based pressure sensor for a high pressure range (up to 1 MPa). **c**, Cyclic response of the CNN-based pressure sensor during 1000 cycles each for 180 kPa, 500 kPa, and 1 MPa. **d**, Enlarged view of the cyclic current response during 1 MPa cycles. **e**, Microscope images of the CNN on a PI film containing a boundary of the pressure applying area before (left) and after (right) the whole cyclic test.

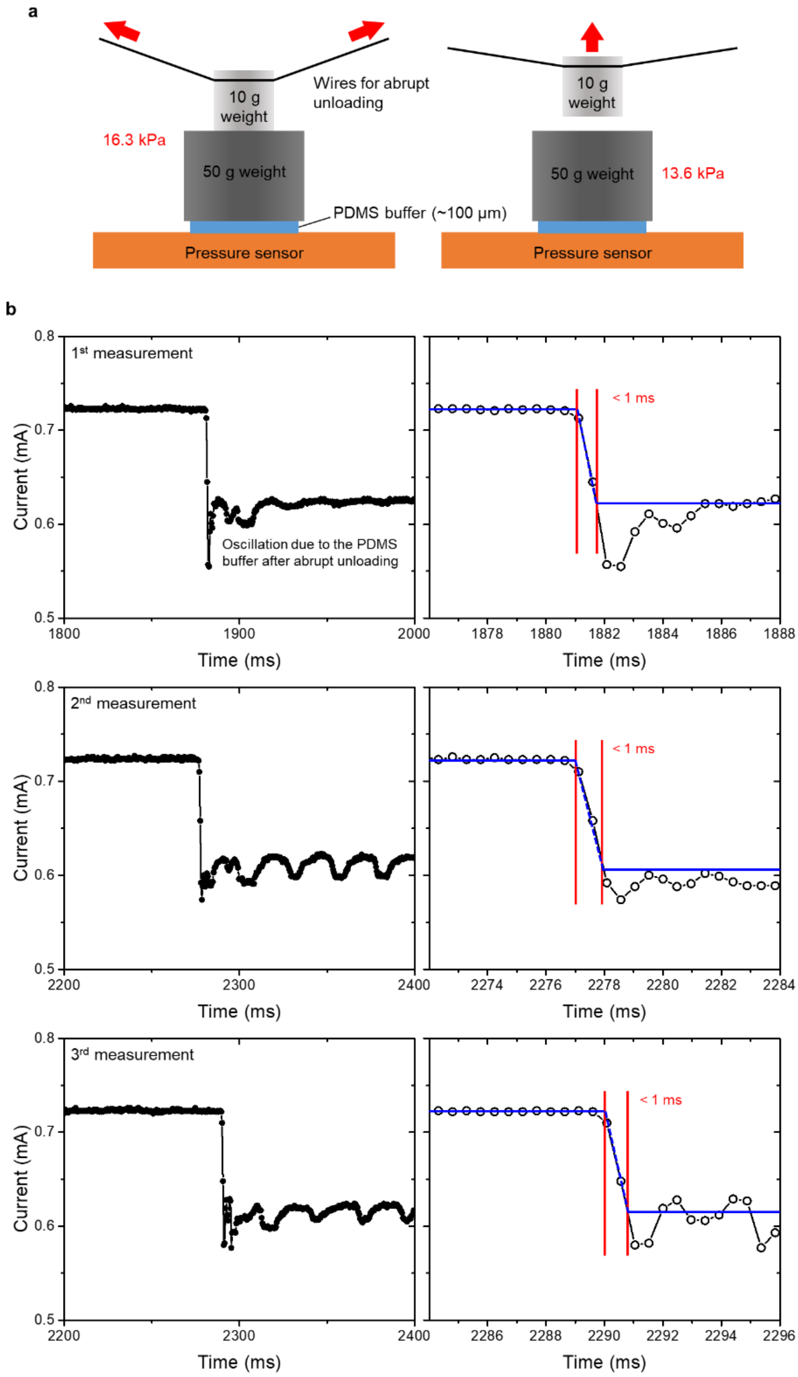

Supplementary Figure 9 | Response time of the CNN. a, Schematic illustration of an abrupt unloading process for response time measurement. It is difficult to make a sudden change in pressure by using the motorized z-stage due to its limited speed. To exclude undesired friction or touch during the unloading process, we used the two wires for unloading a 10 g weight from a 50 g weight. By pulling the two wires in the opposite directions, the 10 g weight was abruptly removed from the 50 g weight, resulting in a sudden pressure decrease from 16.3 kPa to 13.6

kPa. **b**, Responses of the CNN-based pressure sensor to the sudden pressure change. The current was measured at 480-μs intervals. The right graphs are the expanded views of the left three measurement results. Our sensor responded to the sudden pressure decrease, showing an ultrafast response time under 1 ms in the three measurements. After each unloading process, oscillation occurred due to the PDMS buffer and was also captured by our pressure sensor, showing the feasibility of the extremely fast response time.

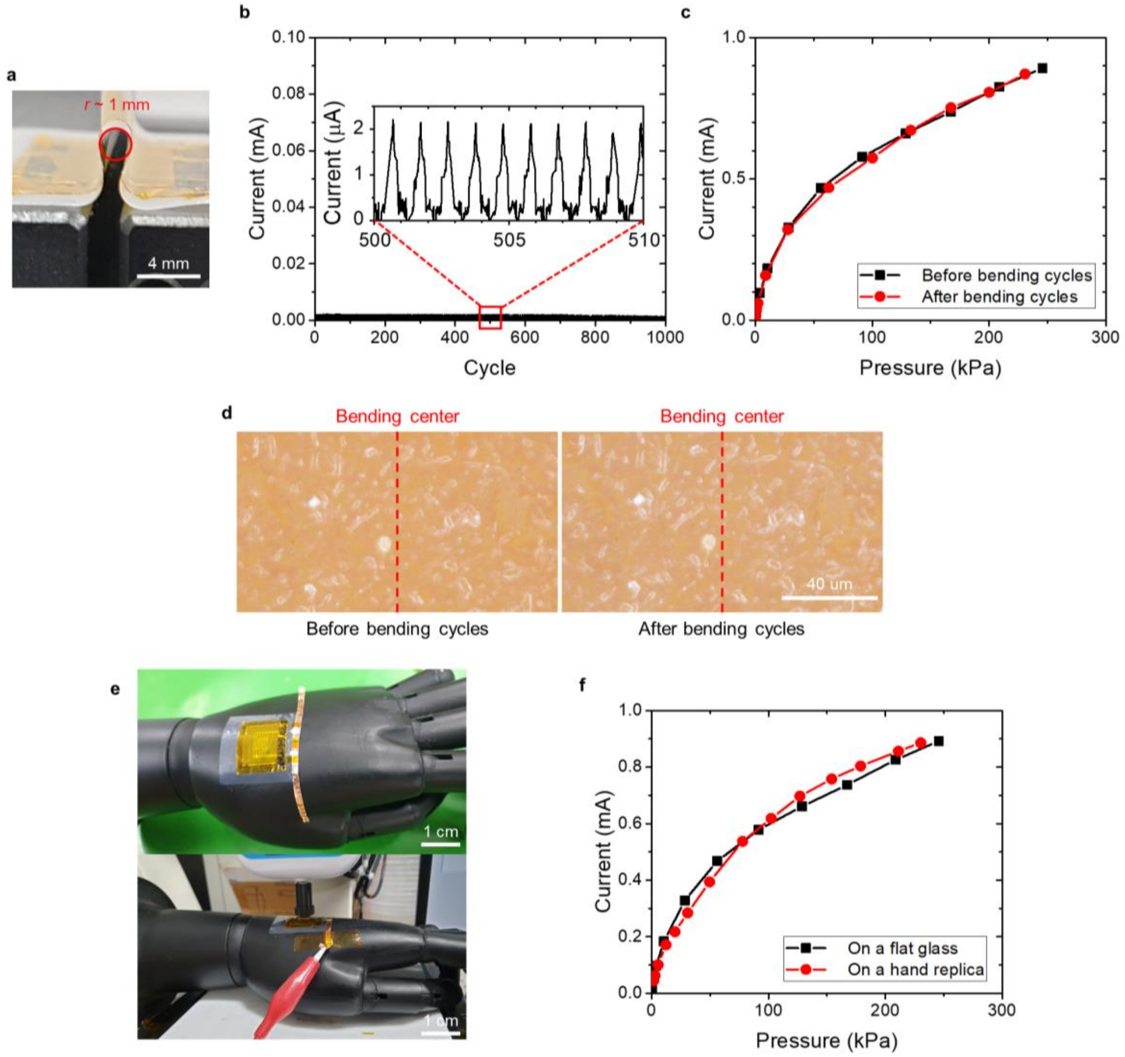

Supplementary Figure 10 | Mechanical reliability of flexible sensors. a, Photograph of the 25-μm-thick sensor that is bent with a bending radius of 1 mm. **b**, Current change of the 25-μm-thick sensor during 1000 bending cycles with a bending radius of 1 mm. **c**, Pressure response of the CNN-based pressure sensor before and after the bending cycles. **d**, Microscope images of the CNN surface before and after the bending cycles. **e**, Optical images of the 25-μm-thick sensor attached to a hand replica. **f**, Pressure response of the 25-μm-thick sensor attached to a hand replica.

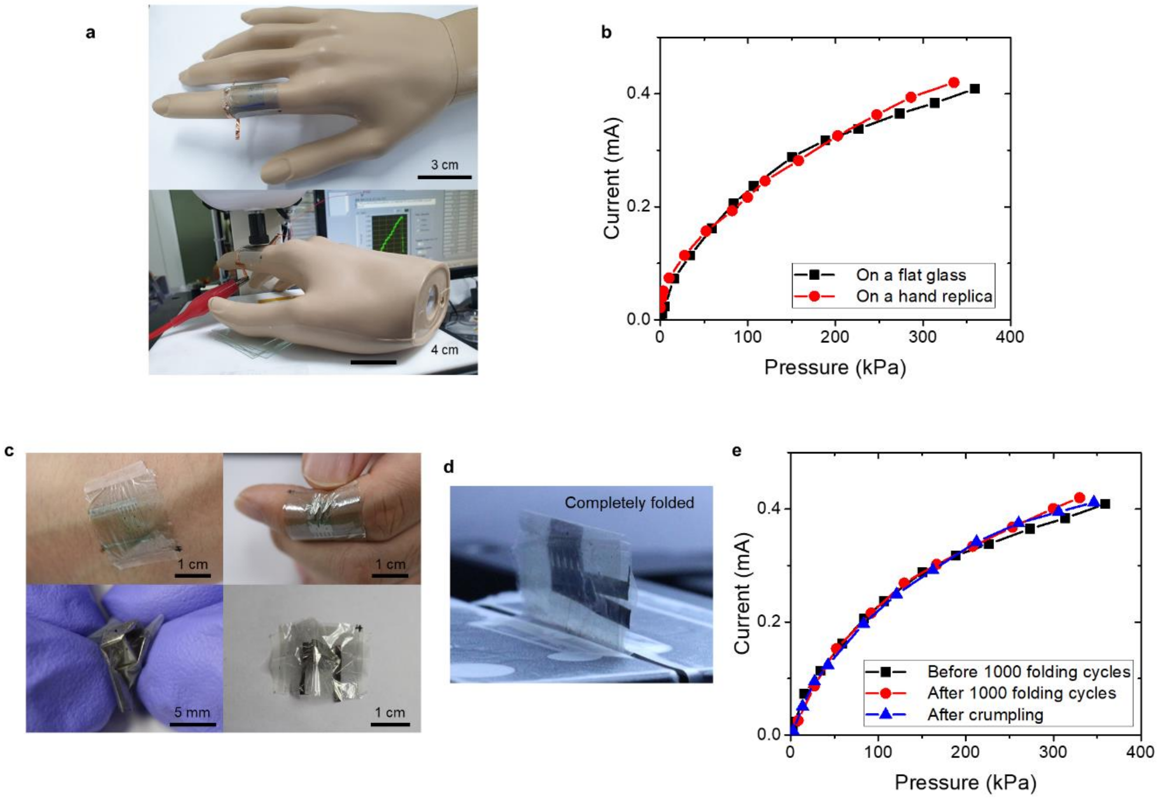

Supplementary Figure 11 | Mechanical reliability of conformable sensors. a, Optical images of the 3-μm-thick sensor attached to a finger of a hand replica. **b**, Pressure response of the 3-μm-thick sensor attached to a finger of a hand replica. **c**, Optical images of the ultrathin sensor under various deformation conditions. **d**, Photograph of the completely folded sensor. **e**, Pressure response of the 3-μm-thick sensor before deformation (black), after 1000 folding cycles (red), and after crumpling (blue).

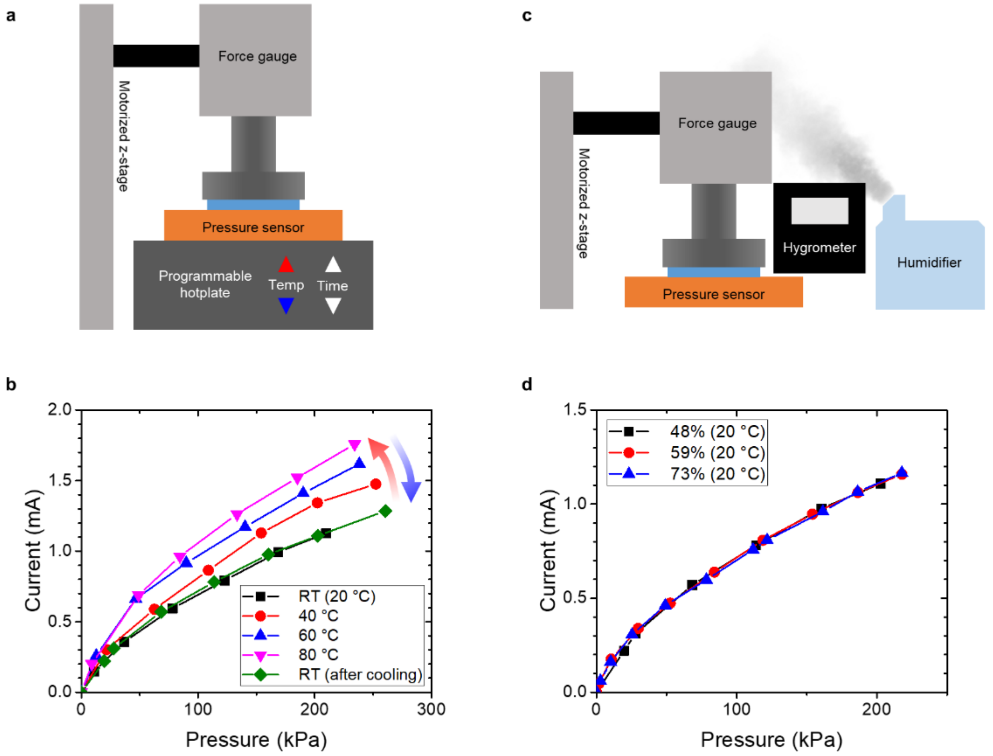

Supplementary Figure 12 | Temperature and humidity dependence. a, Schematic illustration of the experimental setup for investigating the temperature dependence of the sensor performance. **b**, Pressure response of the CNN-based pressure sensor under different temperature conditions. **c**, Schematic illustration of the experimental setup for investigating the humidity dependence of the sensor performance. **d**, Pressure response of the CNN-based pressure sensor under different humidity conditions.

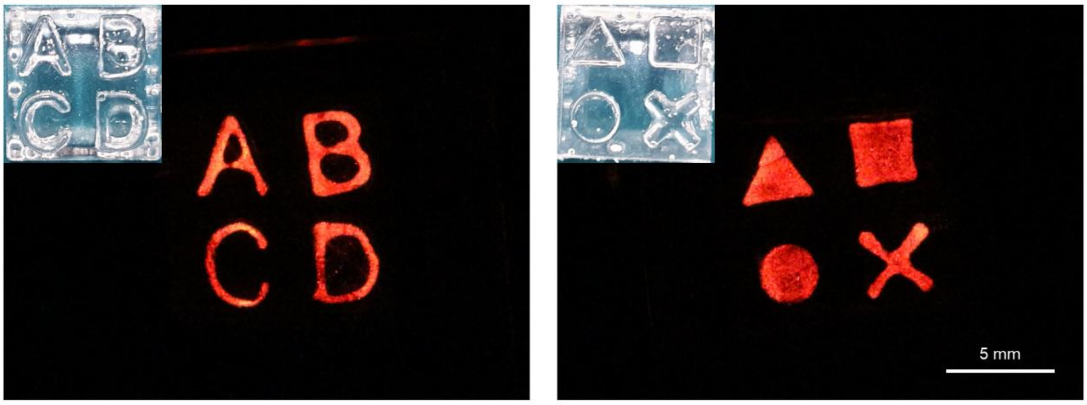

Supplementary Figure 13 | Visualization of the pressure applied by PDMS stamps. The insets show the PDMS stamps. Continuous images representing the precise shapes of the applied stamps were generated. The uneven local light intensity arose from the uneven surface of the PDMS stamps.

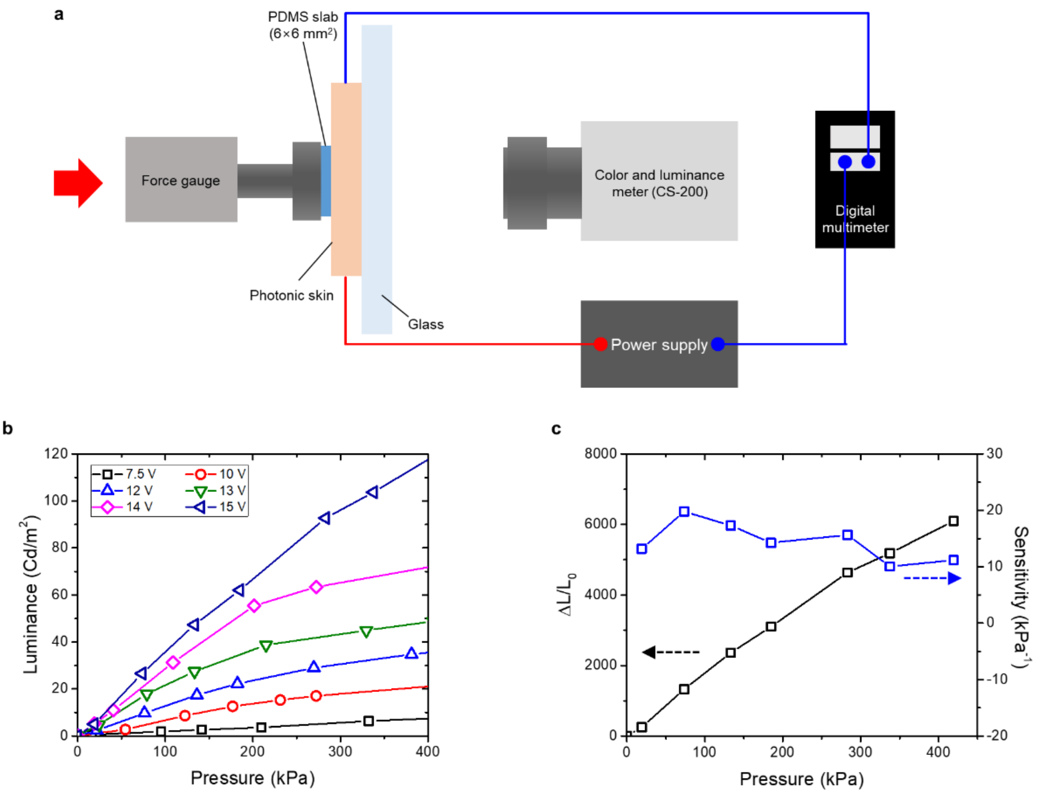

Supplementary Figure 14 | Pressure responses of the photonic skin. a, Schematic illustration of the experimental setup for measuring pressure responses of the photonic skin. **b**, Luminance as a function of the pressure applied with a 6 × 6 mm^2 PDMS slab under various bias voltages. **c**, Ratio of luminance change (ΔL) to initial luminance (L_0) and sensitivity as a function of the applied pressure. The sensitivity was defined as $\delta(\Delta L/L_0)/\delta p$, where p denotes the applied pressure. An initial luminance of 0.02 Cd/m^2 was measured without pressure.

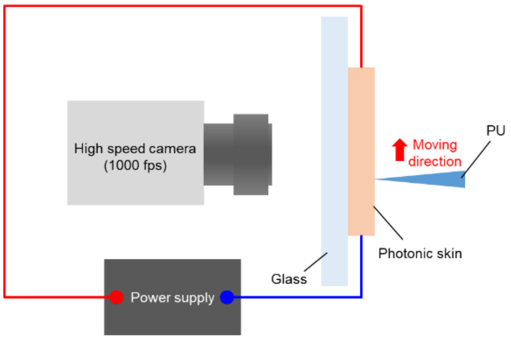

Supplementary Figure 15 | Schematic illustration of the response time measurement. Pressure images were captured using a high-speed camera at 1 ms intervals while a tip of a PU fragment was rapidly sliding on the photonic skin.

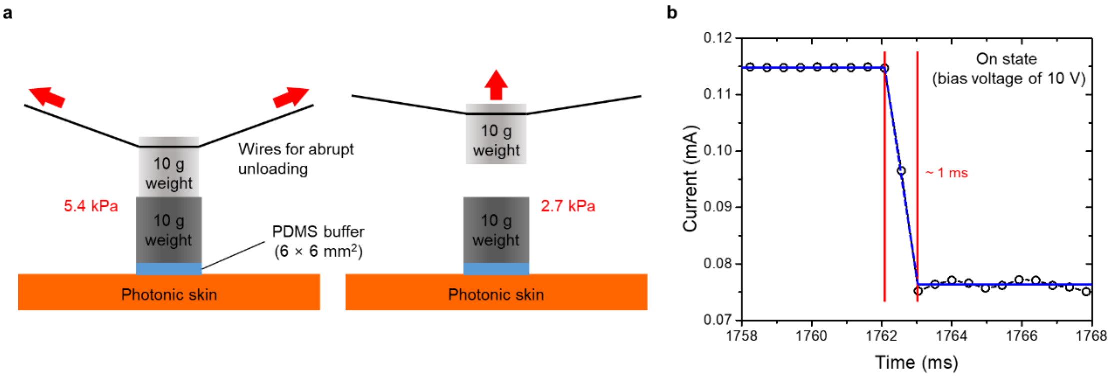

Supplementary Figure 16 | Response time of the photonic skin. a, Schematic illustration of an abrupt unloading process for response time measurement of the photonic skin. **b**, Current response of the photonic skin to the sudden pressure change.

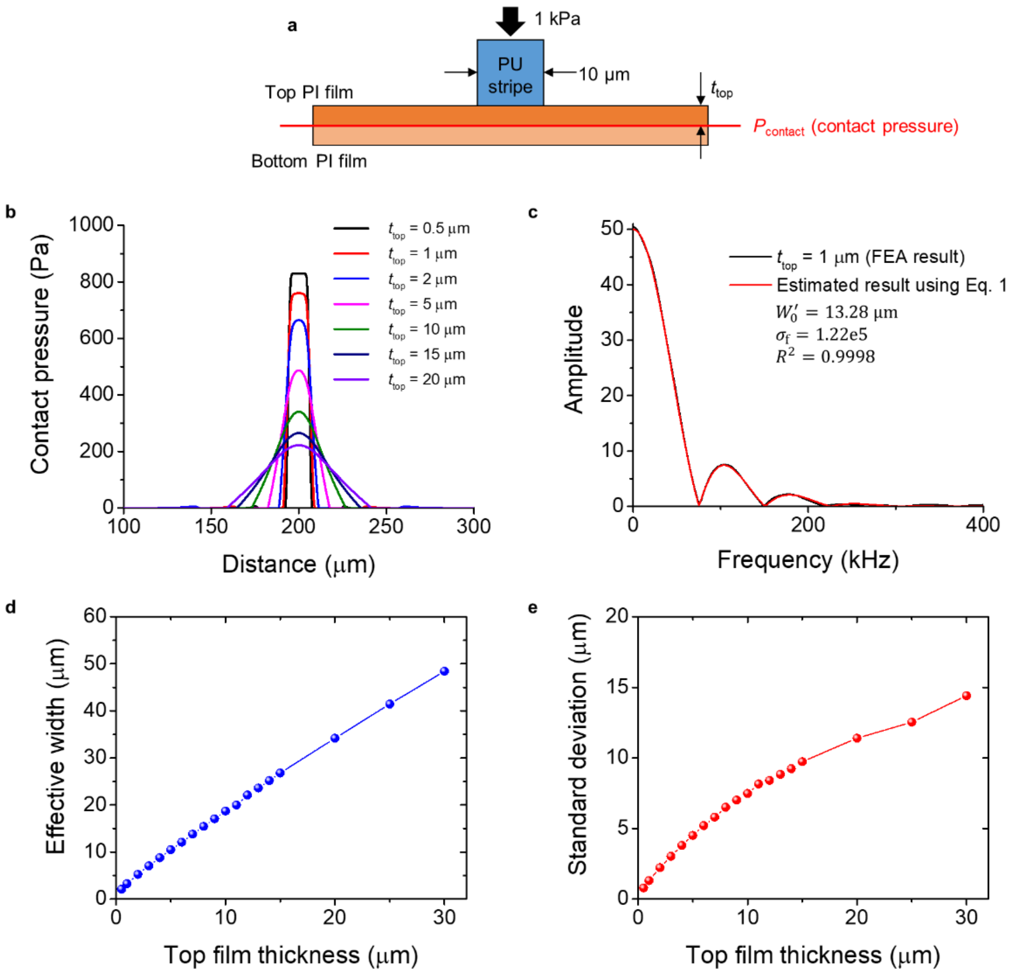

Supplementary Figure 17 | Effect of the film thickness on the output images (2D FEA). a, Modelling details for the 2D FEA simulation. One 10-μm-wide PU stripe pressed the top film with an initial pressure of 1 kPa, and the contact pressure (P_{contact}) between the two films was calculated. **b**, FEA results showing P_{contact} for various t_{top} values. **c**, Comparison between the Fourier transform of the FEA result for $t_{\mathrm{top}} = 1$ μm and the estimated result using Equation 1 in Supplementary Note 2 with $W_0' = 13.28$ μm and $\sigma_{\mathrm{f}} = 1.22e5$. **d** and **e**, W_0' (**d**) and σ_{f} (**e**) as a function of t_{top} with which Equation 1 mostly fits the simulation results.

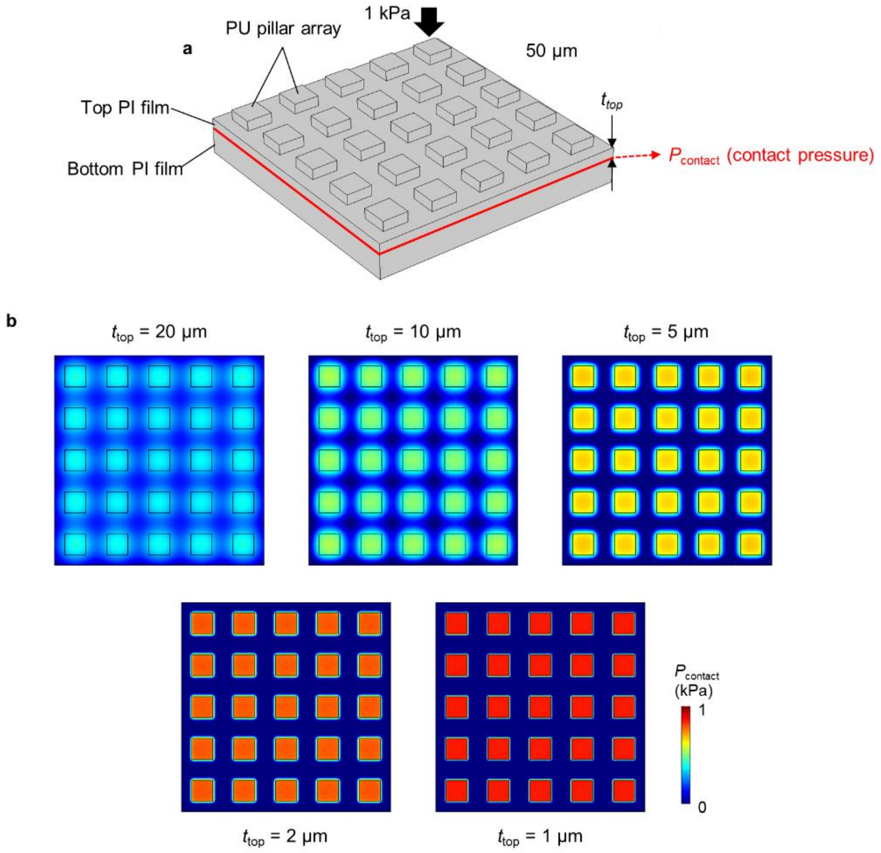

Supplementary Figure 18 | Effect of the film thickness on the output images (3D FEA). a, Modelling details for the 3D FEA simulation. A 5×5 PU pillar with a width of 50 μm and a gap of 50 μm pressed the top film with a uniform initial pressure of 1 kPa, and the contact pressure (P_{contact}) between the two films was calculated. **b**, FEA results showing P_{contact} for various t_{top} values.

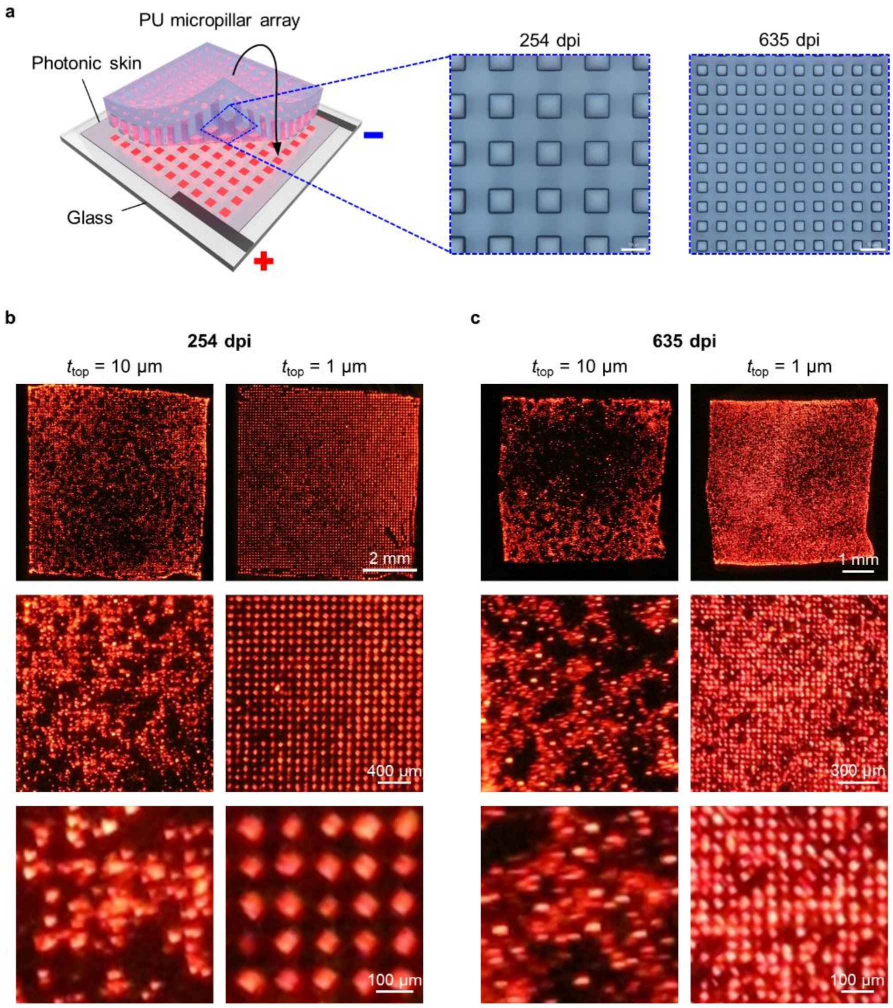

Supplementary Figure 19 | Effect of the top film thickness on the spatial resolution. a, Schematic illustration of the experimental setup for a spatial resolution study using a PU micropillar array. The insets are optical images of the fabricated 254 dpi and 635 dpi PU micropillar arrays. **b**, Optical images where the 254 dpi PU pillar array pressed the photonic skins with t_{top} ~ 10 μm (left column) and t_{top} ~ 1 μm (right column). **c**, Optical images where the 635 dpi PU pillar array pressed the photonic skins with t_{top} ~ 10 μm (left column) and t_{top} ~ 1 μm (right column). The photonic skin with t_{top} ~ 1 clearly displayed the micro-patterned pressure distribution.

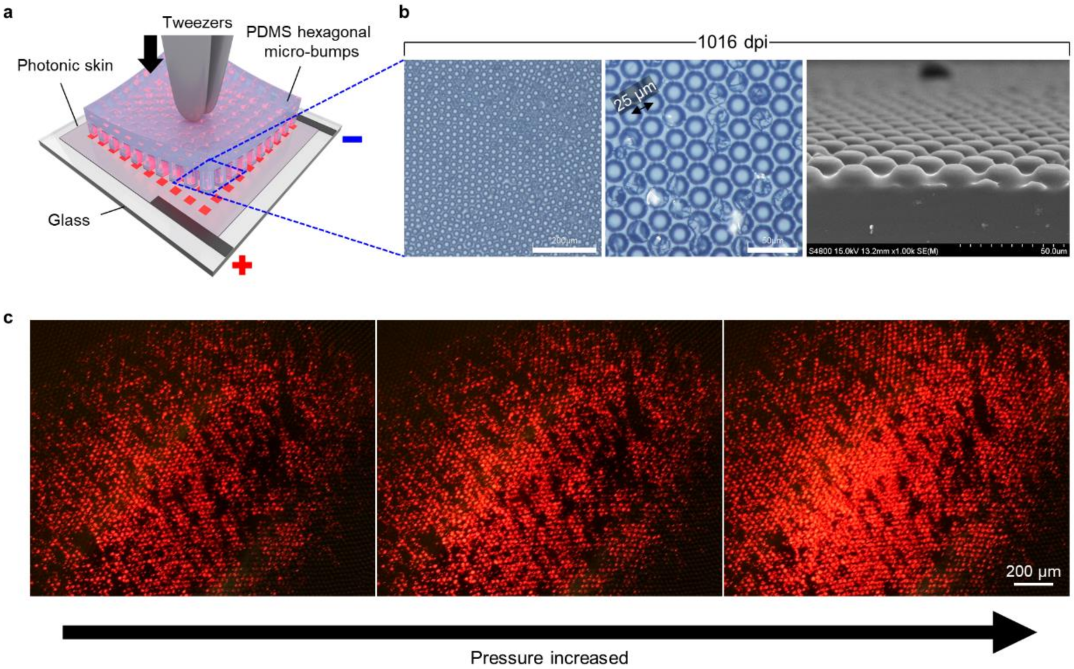

Supplementary Figure 20 | Photonic skin visualizing a 1016 dpi hexagonal texture. a, Schematic illustration of applying focused pressure using PDMS hexagonal micro-bumps and tweezers. We pressed a PDMS micro-bump array on a photonic skin using tweezers. The micro-bump array was fabricated by microsphere lithography using a hexagonally packed monolayer of microsphere (a diameter of 25 μm) as an etching mask (see Methods). **b**, Optical (left and middle) and SEM (right) images of the 1016 dpi micro-bumps. Note that there are a number of defects generated during the fabrication process. **c**, Optical images of the photonic skin visualizing the pressure applied with the 1016 dpi micro-bumps and the tweezers. The device clearly showed both the micro-texture information and large-area pressure distribution concentrated on the tweezer tips. As the force applied with the tweezers increased, the overall light intensity increased.

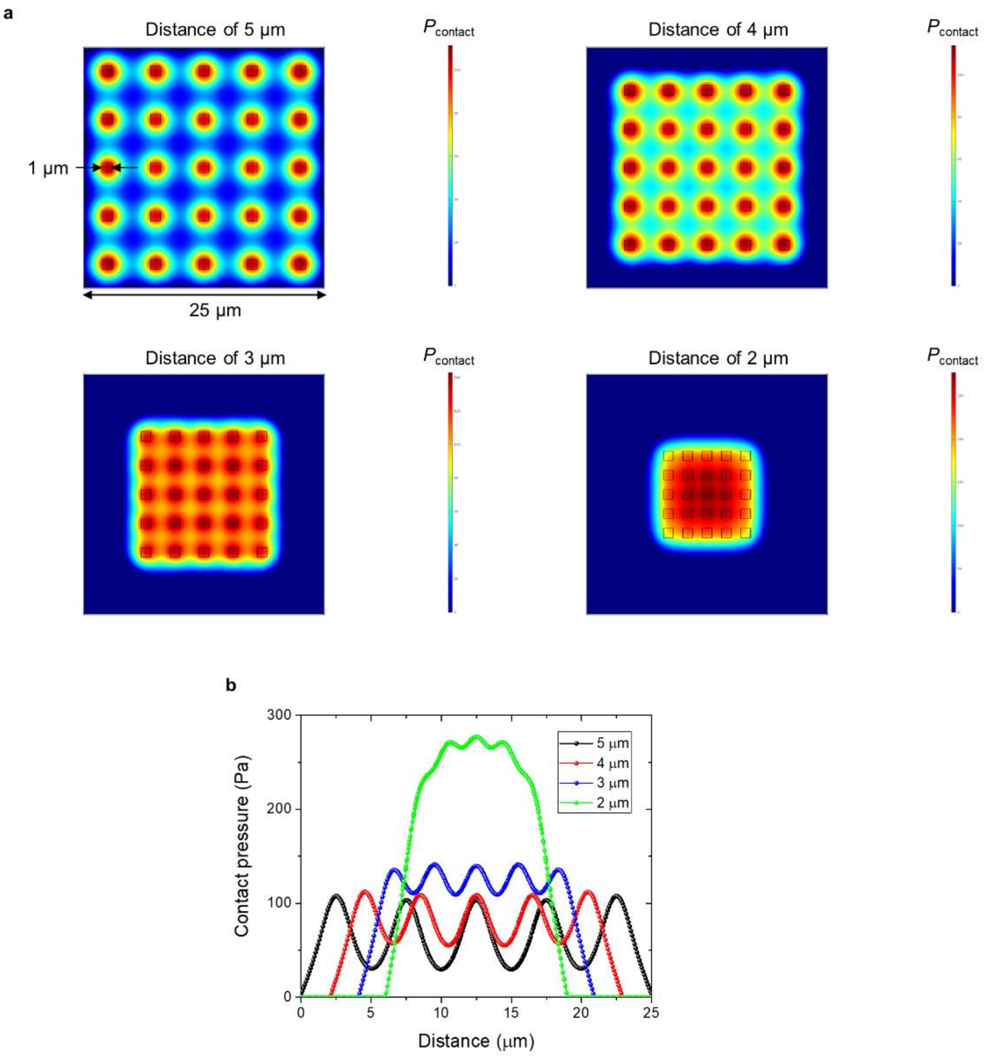

Supplementary Figure 21 | Spatial resolution calculation. **a**, FEA results showing the contact pressure (P_{contact}) between the two films when 1-μm-wide pillars with different distances pressed the top film ($t_{\mathrm{top}} = 1$ μm). As the distance decreases, the patterns are not resolvable in P_{contact}. **b**, Line profiles ($y = 12.5$ μm) for the results in **a**. The minimum distance of 4 μm (6350 dpi) was calculated by the FEA, where the half maximum was located in the middle of the two pillars.

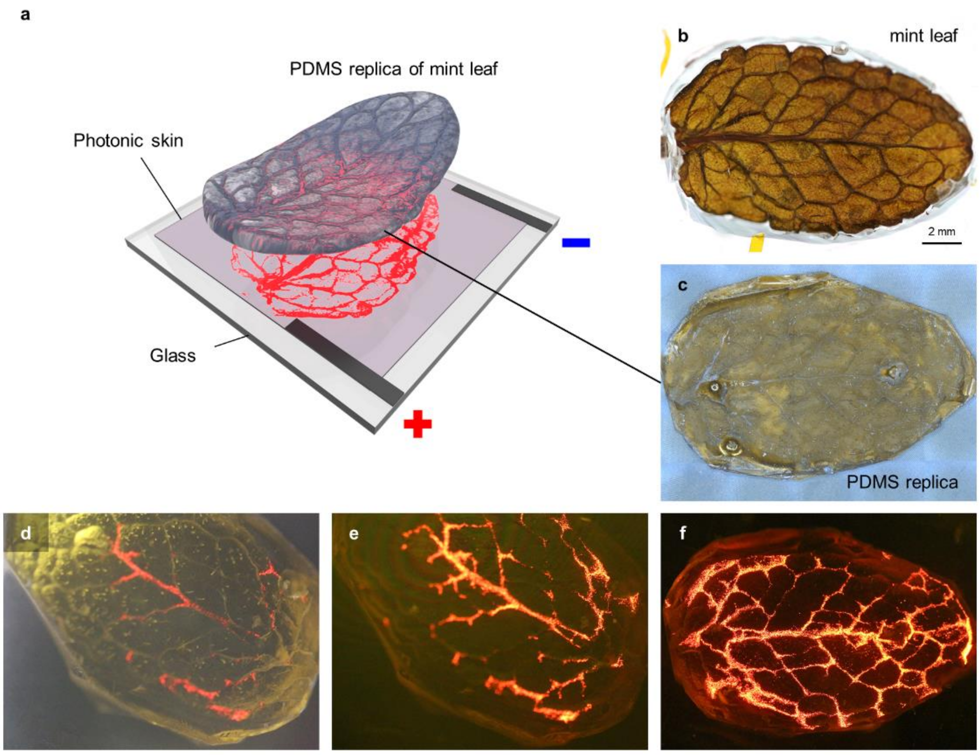

Supplementary Figure 22 | Surface morphology imaging of a mint leaf replica. a, Schematic illustration of the experimental setup for the surface morphology imaging of a mint leaf replica. **b**, Optical photograph of the original mint leaf. **c**, Optical image of the PDMS replica of the mint leaf. **d**, **e** and **f**, Optical photographs of the photonic skin visualizing the surface morphology of the PDMS replica with (**d**) and without (**e** and **f**) ambient light. Because the local pressure applied by the elastic body is proportional to the compressive strain normal to the contact plane, the pressure image of the fully pressed PDMS replica well represents the surface morphology of the mint leaf.

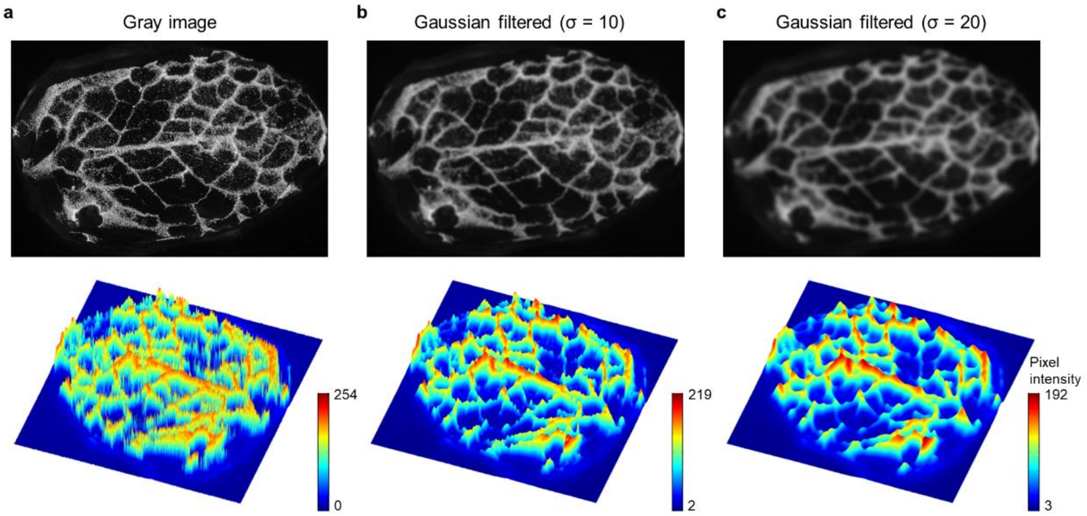

Supplementary Figure 23 | Gaussian-filtered pressure images. a, Greyscale image of the optical pressure image generated by the photonic skin when the mint leaf replica was fully pressed on it (Fig. 5a). **b** and **c**, Gaussian-filtered images of **a** using σ = 10 (**b**) and 20 (**c**). Because of the micro-textures of the mint leaf replica, the captured pressure image contains noisy information representing the local pressure concentrated on the microprotrusions. Gaussian filtering of the pressure image can effectively remove the micro-texture information while maintaining the large-scale morphology of the contact object.

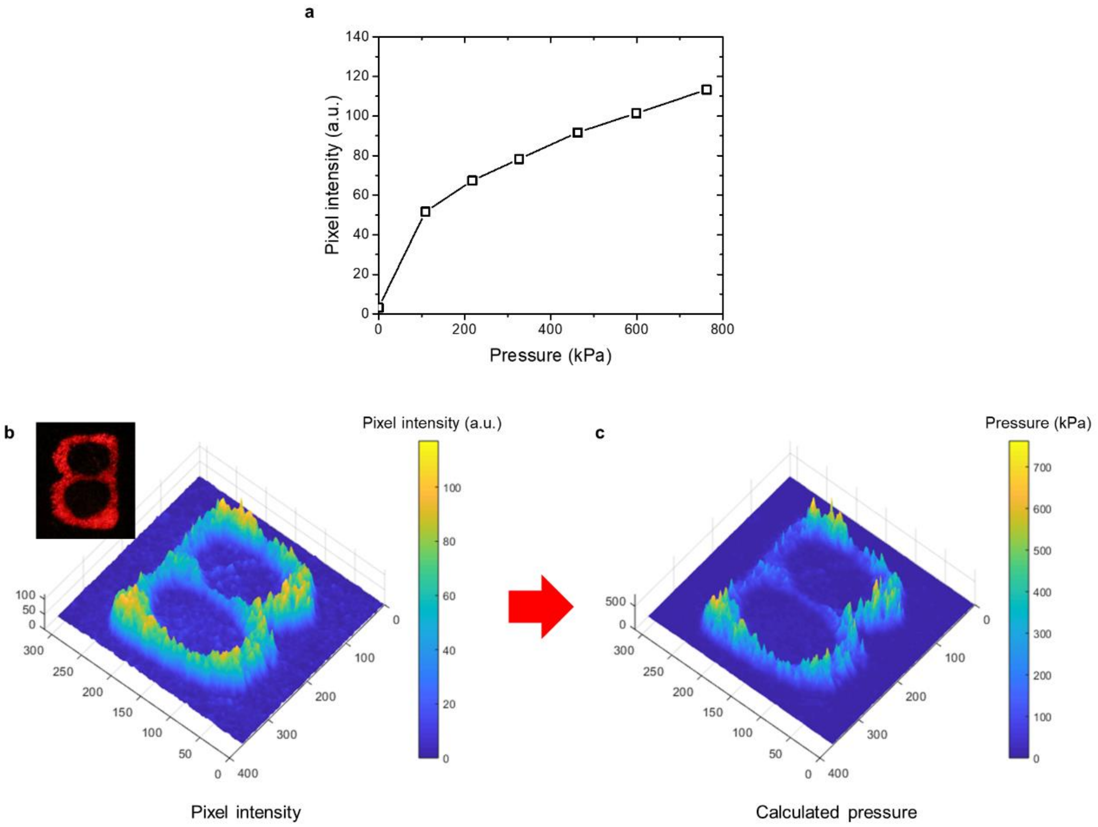

Supplementary Figure 24 | Calculation of pressure distribution. a, Pixel intensity as a function of the pressure applied with a 6 × 6 mm^2 PDMS slab under a bias of 15 V. **b**, Pixel intensity map of the photograph captured by a CCD when the photonic skin was pressed with a B-shaped stamp. **c**, Pressure distribution calculated from the photograph in **b** using the lookup table in **a**. For simplicity, the linear interpolation was used to calculate the pressure values between the measured points.

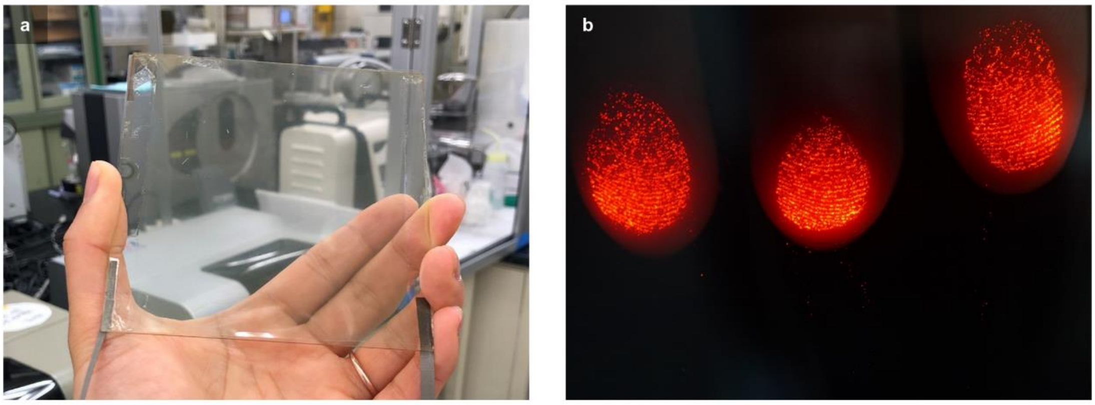

Supplementary Figure 25 | Large-area fabrication of the photonic skin. a, Photograph of a large-area photonic skin fabricated on a glass substrate. **b**, Optical image of the visualization of the pressure applied by multiple fingerprints using the large-area photonic skin.

Supplementary Tables

Reference	Materials and structures	Mechanism	Operating voltage (V)	Sensitivity (kPa^{-1})	Response time (ms)	Detection range (Pa)
1 (2014)	AuNW/tissue paper	Piezoresistive (cellulose)	1.5	1.14 (< 5000 Pa)	< 17	13 – 5000
2 (2017)	SWNT/tissue paper	Piezoresistive (cellulose)	0.1	2.2 (35 – 2500 Pa), 1.3 (2500 – 11700 Pa)	35 – 40	< 11700
3 (2017)	rGO/paper	Piezoresistive (cellulose)		17.2 (1 – 2000), 0.1 (2000 – 20000)	45 – 75	< 20000
4 (2016)	AgNW/cotton fibres	Piezoresistive (cellulose)		3.4 (< 200 Pa)	< 50	< 1000
5 (2017)	CuNW aerogel	Piezoresistive (microstructure)		0.7	80	< 200
6 (2013)	rGO/PU sponge	Piezoresistive (microstructure)		0.26 (< 2000 Pa), 0.03 (2000 – 10000 Pa)		9 – 10000
7 (2014)	PPy foam	Piezoresistive (microstructure)		7.7 – 41.9 (< 100), 0.4 (> 1000)	50	< 100000
8 (2017)	Sparkling graphene block	Piezoresistive (microstructure)	0.1	229 (0 – 120 Pa), 26.9 (400 – 1000 Pa)		10 – 1000
9 (2014)	rGO foam	Piezoresistive (microstructure)		15.2 (< 300 Pa)		165 – 1200
10 (2014)	PEDOT:PSS/PUD micro-pyramid array	Piezoresistive (microstructure)	0.2	10.3		13 – 8000
11 (2014)	CNT/PDMS micro-dome array	Piezoresistive (microstructure)		15.1 (< 500 Pa)	40	0.2 – 70000
12 (2016)	CNT/graphene/PDMS microstructures	Piezoresistive (microstructure)	0.03	19.8 (< 300 Pa)	16.7	0.6 – 6000
13 (2017)	Carbonized silk nanofibre	Piezoresistive (nanofabric)	0.1	34.47 (0.8 – 400 Pa), 1.16 (400 – 5000 Pa)	16.6	0.8 – 5000
14 (2019)	AgNW with air gap	Piezoresistive	10	16.1		< 40000
15 (2018)	Carbonized crepe paper	Piezoresistive (fabric)	1	2.56 - 5.67 (0 – 2530 Pa)	30	0.9 – 20000
This work	CNN	Piezoresistive (nanocellulose)	1	> 10000 (< 10000 Pa), ~5000 (10000 – 170000 Pa)	< 1	< 170000

Supplementary Table 1 | Performance comparison of piezoresistive pressure sensors.

Reference	Mapping type	Mechanism	Sensing type	Working range	Sensitivity	Spatial resolution	Spatial fill factor	Response time
16 (2013)	Electrical Mapping	PSR and TFTs	Touch	-	-	~4 dpi (pixelated)	-	-
7 (2014)	Electrical Mapping	Piezoresistive sensors	Real-time pressure	0.8 Pa – 10 kPa	133.1 kPa^{-1}	~4.2 dpi (pixelated)	-	< 50 ms
17 (2019)	Electrical Mapping	Piezoresistive sensors	Real-time pressure	0 Pa – 200 kPa	0.97 kPa^{-1}	~5.1 dpi (pixelated)	-	24 ms
18 (2010)	Electrical Mapping	PSR and NW TFTs	Real-time pressure	0 Pa – 15 kPa	~11.5 μS kPa^{-1}	~10 dpi (pixelated)	-	< 100 ms
19 (2013)	Electrical mapping	Triboelectric sensors	Real-time pressure	2.1 Pa – 40 kPa	0.31 kPa^{-1}	~1 dpi (pixelated)	-	< 5 ms
20 (2016)	Electrical mapping	Triboelectric sensors	Real-time pressure	1 kPa – 150 kPa	0.06 kPa^{-1}	5 dpi (pixelated)	-	70 ms
21 (2017)	Electrical mapping<br>Mechanoluminescent imaging	Triboelectric sensors (low pressure)<br>Piezoelectric fluorescence (high pressure)	Real-time pressure (low pressure)<br>Pressure change (high pressure)	60 Pa – 200 kPa<br>600 kPa – 20 Mpa	0.006 kPa^{-1}<br>0.000037 kPa^{-1}	100 dpi (pixelated)	-	50 ms<br>~9 ms
22 (2013)	Electroluminescent Imaging	Pixelated OLED, TFT, and PSR	Real-time pressure	10 kPa – 100 kPa	42.7 Cd m^{-2} kPa^{-1}	~14 dpi (pixelated)	~44%	< 100 ms
23 (2013)	Electroluminescent Imaging	Piezoelectric nanowire LEDs	Real-time pressure	~1 MPa –	12.88 GPa^{-1}	6350 dpi (pixelated)	11%	~90 ms
24 (2015)	Electroluminescent Imaging	Piezoelectric nanowire LEDs	Real-time pressure	~1 MPa –	39.09 GPa^{-1}	6350 dpi (pixelated)	3%	-
25 (2015)	Mechanoluminescent imaging	Piezoelectric fluorescence	Pressure change	1 MPa – 50 MPa	2.2 cps kPa^{-1}	254 dpi (pixelated)	25%	10 ms
This work	Electroluminescent Imaging	Ultrathin layers of CNN and QLED	Real-time pressure	1 kPa – 1 MPa	10 kPa^{-1}	> 1000 dpi (continuous)	~100%	1 ms

Supplementary Table 2 | Comparison of pressure mapping devices. The limitations of each technology are highlighted in red.

Supplementary Notes

Supplementary Note 1 | Spatial resolution of the photonic skin. To investigate the effect of the top film thickness (t_{top}) on the spatial resolution of the photonic skin, we carried out a systematic finite element analysis (FEA) calculating the contact pressure (P_{contact}) between the top and bottom films when spatially patterned input pressure (P_{input}) was applied to the top film (Fig. 4, Supplementary Fig. 17, 18, and 21).

Supplementary Note 2 | Theoretical postulation. Assume that a rectangular pulse (width of W_0 and amplitude of P_0) of spatial pressure was applied to the top surface of the top film with a thickness of t_{top}. If the top film is thin compared to W_0, it will conform to the applied pressure. The bottom film feels as if a rectangular pulse with a larger width and rounded edges touches it, which can be seen as spatial low-pass filtering of a larger pulse. If the low-pass filter is assumed to be a Gaussian filter, the spatial Fourier transform of P_{contact} can be expressed as

$$P_{W_0', \sigma_{\mathrm{f}}}(f) = P_0' \cdot W_0' \cdot \mathrm{sinc}(W_0' f) \cdot g_{\sigma_{\mathrm{f}}}(f) \qquad (1)$$

where W_0' is the increased width of the larger pulse (effective width), P_0' is the reduced amplitude of the pulse due to the increased width, and $g_{\sigma_{\mathrm{f}}}(f)$ is a normal probability density function with a mean of 0 and a standard deviation of σ_{f}. If we compare the estimated values using Equation 1 with the FEA results for different t_{top} values and determine σ_{f} and W_0' for a specific t_{top}, we can quantitatively explain the effect of t_{top} on the output images.

Supplementary Note 3 | 2D FEA. We carried out a 2D FEA, where one 10-µm-wide PU stripe pressed the top film, as depicted in Supplementary Fig. 17a. As we expected, the P_{contact}

becomes a blurred form of P_{input} as t_{top} increases (Supplementary Fig. 17b). In the case of $W_0 = 10$ µm and $t_{\text{top}} = 1$ µm, the value estimated using Equation 1 with $W_0' = 13.28$ µm and $\sigma_{\text{f}} = 1.22e5$ well fit the simulation result with $R^2 = 0.9998$ (Supplementary Fig. 17c). We found the W_0' and σ_{f} with which Equation 1 mostly fits the simulation results and plotted them as a function of t_{top} (Supplementary Fig. 17d and e). The graphs reveal that a thicker top film results in a larger W_0' (thus smaller P_0') and a smaller σ_{f} corresponding to a narrower passband in the low-pass filtering. These results quantitatively explain that the masking of the top film acts as a spatial low-pass filter, where the passband is inversely proportional to the thickness of the masking film.

Supplementary Note 4 | 3D FEA and experimental results. Figure 4c and Supplementary Fig. 18 show the 3D FEA results obtained when a pillar array with a 50 µm width and a 50 µm gap (254 dpi) pressed the top film, further supporting this "masking effect" of the top film. Modelling details of the FEA are illustrated in Supplementary Fig. 18a. As we increased the t_{top} in the FEA, P_{contact} at the contact plane became a blurred form of P_{input}. We also experimentally verified the FEA results using 254 dpi and 635 dpi PU micropillar arrays and photonic skins with $t_{\text{top}} \sim 1$ µm and $t_{\text{top}} \sim 10$ µm (Fig. 4d, e and Supplementary Fig. 19). Figure 4d and the left column in Supplementary Fig. 19b show the experimental results obtained when the photonic skin with $t_{\text{top}} \sim 10$ µm was pressed by the 254 dpi pillar array. It generated a distorted and collapsed pressure image containing the large-area topography of the pillar array due to the uneven pillar height and noise due to the top film roughness. On the other hand, the device with $t_{\text{top}} \sim 1$ µm displayed a high-quality and distinct image of the spatial pressure patterned with the 254 dpi pillar array (Fig. 4e and the right column in Supplementary

Fig. 19b). The experimental results show good agreement with the FEA results. Furthermore, the photonic skin with $t_{\mathrm{top}} \sim 1\ \mu\mathrm{m}$ even clearly visualized the pressure applied with a 635 dpi micropillar array (Supplementary Fig. 19c) and a 1016 dpi hexagonal micro-bump array (Fig. 4f and Supplementary Fig. 20).

Supplementary Note 5 | Spatial resolution calculation for $\boldsymbol{t_{top}} = \mathbf{1\ \mu m}$. The spatial resolution in analogue imaging that does not have pixels, such as our photonic skin, can be defined as the minimum distance between two impulses that are resolvable. The impulse function in the spatial pressure is difficult to realize both in the FEA and experimentally. Therefore, we calculated the spatial resolution for 1-μm-wide pillars and the photonic skin with $t_{\mathrm{top}} = 1$ μm. The FEA results are shown in Supplementary Fig. 21. A minimum distance of 4 μm (6350 dpi) was calculated by the FEA, where the half maximum is located in the middle of the two pillars, as shown by the line profiles in Supplementary Fig. 21b. By using our experimental setup, we were not able to obtain experimental evidence for this calculation, but we do note that the spatial resolution of our device far surpasses the experimentally measured value (1016 dpi), which could be observed by using higher-resolution micropillars and constructing an appropriate measuring system.

Supplementary References

1. Gong, S. *et al.* A wearable and highly sensitive pressure sensor with ultrathin gold nanowires. *Nat. Commun.* **5,** 3132 (2014).
2. Zhan, Z. *et al.* Paper/Carbon Nanotube-Based Wearable Pressure Sensor for Physiological Signal Acquisition and Soft Robotic Skin. *ACS Appl. Mater. Interfaces* **9,** 37921–37928 (2017).
3. Tao, L.-Q. *et al.* Graphene-Paper Pressure Sensor for Detecting Human Motions. *ACS Nano* **11,** 8790–8795 (2017).
4. Wei, Y., Chen, S., Lin, Y., Yuan, X. & Liu, L. Silver nanowires coated on cotton for flexible pressure sensors. *J. Mater. Chem. C* **4,** 935–943 (2016).
5. Xu, X. *et al.* Copper Nanowire-Based Aerogel with Tunable Pore Structure and Its Application as Flexible Pressure Sensor. *ACS Appl. Mater. Interfaces* **9,** 14273–14280 (2017).
6. Yao, H.-B. *et al.* A Flexible and Highly Pressure-Sensitive Graphene-Polyurethane Sponge Based on Fractured Microstructure Design. *Adv. Mate.* **25,** 6692–6698 (2013).
7. Pan, L. *et al.* An ultra-sensitive resistive pressure sensor based on hollow-sphere microstructure induced elasticity in conducting polymer film. *Nat. Commun.* **5,** 3002 (2014).
8. Lv, L., Zhang, P., Xu, T. & Qu, L. Ultrasensitive Pressure Sensor Based on an Ultralight Sparkling Graphene Block. *ACS Appl. Mater. Interfaces* **9,** 22885–22892 (2017).
9. Hou, C., Wang, H., Zhang, Q., Li, Y. & Zhu, M. Highly Conductive, Flexible, and Compressible All-Graphene Passive Electronic Skin for Sensing Human Touch, *Adv. Mater.* **26**, 5018-5024 (2014)
10. Choong, C.-L. *et al.* Highly Stretchable Resistive Pressure Sensors Using a Conductive Elastomeric Composite on a Micropyramid Array. *Adv. Mater.* **26,** 3451–3458 (2014).
11. Park, J. *et al.* Giant Tunneling Piezoresistance of Composite Elastomers with Interlocked Microdome Arrays for Ultrasensitive and Multimodal Electronic Skins. *ACS Nano* **8,** 4689–4697 (2014).
12. Jian, M. *et al.* Flexible and Highly Sensitive Pressure Sensors Based on Bionic Hierarchical Structures. *Adv. Funct. Mater.* **27,** 1606066 (2017).
13. Wang, Q., Jian, M., Wang, C. & Zhang, Y. Carbonized Silk Nanofiber Membrane for Transparent and Sensitive Electronic Skin. *Adv. Funct. Mater.* **27,** 1605657 (2017).
14. Jeong, H., Noh, Y., Ko, S. H. & Lee, D. Flexible resistive pressure sensor with silver nanowire networks embedded in polymer using natural formation of air gap. *Compos. Sci. Technol.* **174,** 50–57 (2019).
15. Chen, S., Song, Y. & Xu, F. Flexible and Highly Sensitive Resistive Pressure Sensor Based on Carbonized Crepe Paper with Corrugated Structure. *ACS Appl. Mater. Interfaces* **10,** 34646–34654 (2018).
16. Kaltenbrunner, M. *et al.* An ultra-lightweight design for imperceptible plastic electronics. *Nature* **499,** 458–463 (2013).

17. Kim, K. K. *et al.* Transparent wearable three-dimensional touch by self-generated multiscale structure. *Nat. Commun.* **10,** 2582 (2019).

18. Takei, K. *et al.* Nanowire active-matrix circuitry for low-voltage macroscale artificial skin. *Nat. Mater.* **9,** 821–826 (2010).

19. Lin, L. *et al.* Triboelectric Active Sensor Array for Self-Powered Static and Dynamic Pressure Detection and Tactile Imaging. *ACS Nano* **7,** 8266–8274 (2013).

20. Wang, X. *et al.* Self-Powered High-Resolution and Pressure-Sensitive Triboelectric Sensor Matrix for Real-Time Tactile Mapping. *Adv. Mater.* **28,** 2896–2903 (2016).

21. Wang, X. *et al.* Full Dynamic-Range Pressure Sensor Matrix Based on Optical and Electrical Dual-Mode Sensing. *Adv. Mater.* **29,** 1605817 (2017).

22. Wang, C. *et al.* User-interactive electronic skin for instantaneous pressure visualization. *Nat. Mater.* **12,** 899–904 (2013).

23. Pan, C. *et al.* High-resolution electroluminescent imaging of pressure distribution using a piezoelectric nanowire LED array. *Nat. Photonics* **7,** 752–758 (2013).

24. Peng, M. *et al.* High-Resolution Dynamic Pressure Sensor Array Based on Piezo-phototronic Effect Tuned Photoluminescence Imaging. *ACS Nano* **9,** 3143–3150 (2015).

25. Wang, X. *et al.* Dynamic Pressure Mapping of Personalized Handwriting by a Flexible Sensor Matrix Based on the Mechanoluminescence Process. *Adv. Mater.* **27,** 2324–2331 (2015).
