# Supplementary material for: Ultraflexible and transparent electroluminescent skin for real-time and super-resolution imaging of pressure distribution
Source: Nat Commun. 2020 Jan 31;11:663. doi: 10.1038/s41467-020-14485-9 (PMC6994701; doi:10.1038/s41467-020-14485-9)
Supplement: Supplementary file 2 — Description of Additional Supplementary Information [file 41467_2020_14485_MOESM2_ESM.docx]

**Description of Additional Supplementary Files**

**File Name:** Supplementary Movie 1
**Description:** Response time of the photonic skin. Pressure images were captured using a high-speed camera at 1000 fps while a tip of a PU fragment was rapidly sliding on the photonic skin. The captured images were played back at 30 fps, so the playback speed is 0.03 times. Each frame captured at 1 ms intervals showed the exact touch position of the PU tip without any trace or delay, revealing that our photonic skin has a response time of < 1 ms.

**File Name:** Supplementary Movie 2
**Description:** Imaging of pressure applied with a PDMS replica of a mint leaf. A real-time pressure image was recorded while a PDMS replica of a mint leaf was repeatedly pressed on the device with a hand. Because of the high sensitivity and linearity of the photonic skin, the high-contrast pressure image well represented the surface morphology of the compressed part of the leaf replica. The video also shows the fast response time and cyclic stability of the photonic skin.

**File Name:** Supplementary Movie 3
**Description:** Operation of a photonic skin laminated on a hand. A photonic skin was laminated on a hand and touched with a finger and a glass rod. The device showed touch force and position in real time.

**File Name:** Supplementary Movie 4
**Description:** Real-time touch sensing with fingerprint recognition. Our fast and high-resolution pressure imaging can be utilized for advanced touch interfaces capable of identifying the user in addition to sensing the touch force and location. For a proof of concept, the pressure images were recorded by a charge-coupled device (CCD) at 15 fps and transferred to a computer, while the photonic skin was touched with a finger. A fingerprint was then recognized using a template matching algorithm and denoted by a rectangle in real time.
